# Kinase-independent role of cyclin D1 in chromosomal instability and mammary tumorigenesis

**DOI:** 10.18632/oncotarget.3267

**Published:** 2015-03-25

**Authors:** Mathew C. Casimiro, Gabriele Di Sante, Marco Crosariol, Emanuele Loro, William Dampier, Adam Ertel, Zuoren Yu, Elizabeth A. Saria, Alexandros Papanikolaou, Zhiping Li, Chenguang Wang, Sankar Addya, Michael P. Lisanti, Paolo Fortina, Robert D. Cardiff, Aydin Tozeren, Erik S. Knudsen, Andrew Arnold, Richard G. Pestell

**Affiliations:** ^1^ Departments of Cancer Biology, Thomas Jefferson University & Hospital, Philadelphia, PA 19107, USA; ^2^ Medical Oncology, Thomas Jefferson University & Hospital, Philadelphia, PA 19107, USA; ^3^ Stem Cell and Regenerative Medicine, Thomas Jefferson University & Hospital, Philadelphia, PA 19107, USA; ^4^ Sidney Kimmel Cancer Center, Philadelphia, PA 19107, USA; ^5^ Center for Integrated Bioinformatics, School of Biomedical Engineering, Science and Health Systems, Drexel University, Philadelphia, PA 19104, USA; ^6^ Center for Molecular Medicine, University of Connecticut Health Center, Farmington, CT 06030, USA; ^7^ Department of Pathology and Laboratory Medicine, UC Davis Center for Comparative Medicine, University of California, Davis CA 95616, USA; ^8^ Kazan Federal University, Kazan 420008, Republic of Tatarstan, Russian Federation

**Keywords:** Cyclin D1, breast cancer, chromosomal instability

## Abstract

Cyclin D1 is an important molecular driver of human breast cancer but better understanding of its oncogenic mechanisms is needed, especially to enhance efforts in targeted therapeutics. Currently, pharmaceutical initiatives to inhibit cyclin D1 are focused on the catalytic component since the transforming capacity is thought to reside in the cyclin D1/CDK activity. We initiated the following study to directly test the oncogenic potential of catalytically inactive cyclin D1 in an *in vivo* mouse model that is relevant to breast cancer. Herein, transduction of *cyclin D1^−/−^* mouse embryonic fibroblasts (MEFs) with the kinase dead KE mutant of cyclin D1 led to aneuploidy, abnormalities in mitotic spindle formation, autosome amplification, and chromosomal instability (CIN) by gene expression profiling. Acute transgenic expression of either *cyclin D1^WT^* or *cyclin D1^KE^* in the mammary gland was sufficient to induce a high CIN score within 7 days. Sustained expression of *cyclin D1^KE^* induced mammary adenocarcinoma with similar kinetics to that of the wild-type cyclin D1. ChIP-Seq studies demonstrated recruitment of cyclin D1^WT^ and cyclin D1^KE^ to the genes governing CIN. We conclude that the CDK-activating function of cyclin D1 is not necessary to induce either chromosomal instability or mammary tumorigenesis.

## INTRODUCTION

Activation of the *cyclin D1* oncogene, often by amplification or rearrangement, is a major driver of multiple types of human tumors including breast and squamous cell cancers, B-cell lymphoma, myeloma, nd parathyroid adenoma [1, 2]. The *cyclin D1* gene is amplified or overexpressed in up to half of human breast cancers and its mammary-targeted overexpression induces mammary tumorigenesis in mice [3]. *Cyclin D1* encodes the regulatory subunit of the cyclin-dependent kinase (CDK4/6) holoenzyme. Tumors overexpressing cyclin D1 tend to display normal levels of proliferation and expression of E2F target genes, which contrasts with tumors overexpressing cyclin E or an activator for pRb [4, 5]. Breast cancers overexpressing cyclin D1 that are wild type for *pRb* have relatively normal proliferation rates, in contrast to those caused by genetic inactivation of *pRb*, which show significantly increased proliferation rates [4–6]. Furthermore, the alternate splice form of cyclin D1, (cyclin D1b), has potent transforming ability, which does not correlate with the ability to phosphorylate the pRb protein [7, 8].

Much of the early work defined kinase-dependent functions of cyclin D1 (reviewed in [9]). Cyclin D1/CDK4/6 phosphorylates the retinoblastoma protein (pRb) to advance the G_1_S and phosphorylates NRF-1 to inhibit mitochondrial biogenesis thereby coordinating nuclear and mitochondrial functions [10–13]. Cyclin D1 regulates a pool of mammary progenitor cells (parity-identified mammary cells: PI-MEC) is kinase-dependent. The resistance of *cyclin D1^−/−^*/**MMTV-ErbB2 mice to ErbB2 driven mammary tumors is thought to be dependent on a complete absence of PI mammary cells in cyclin D1-null mice [14]. Several other kinase-dependent properties of cyclin D1 have been identified including the induction of cellular migration, enhanced angiogenesis and mammary stem cell self-renewal [15–17].

In addition to the function of cyclin D1 as a regulatory subunit of a CDK holoenzyme, several CDK independent functions have been identified. Cyclin D1 also functions as a transcriptional regulator, usually in a CDK4-independent manner [8]. Cyclin D1 also mediates DNA-damage repair signaling in a CDK4-independent manner [18]. Chromatin immunoprecipitation studies identified cyclin D1 in the context of local chromatin, and the abundance of cyclin D1 determined the recruitment of transcription factors (TF) [19]. The recruitment of cyclin D1 to *cis* elements enriches for histone acetylases (p300/CBP), histone deacetylases, the methylase SUV39 and the heterochromatin protein HP1α in ChIP [20]. ChIP-ChIP demonstrated cyclin D1 and p300 together occupied genes in close proximity to the transcriptional start site [21], and whole genome ChIP-Seq demonstrated enrichment of cyclin D1 at genes that regulate mitosis and chromosomal stability [22]. In MEFs and in transgenic mice cyclin D1 induced chromosomal instability (CIN) gene expression. CIN occurs frequently in tumors [23] and is characterized by altered rates of loss or gain of whole chromosomes and/or structural chromosomal aberrations [24]. However, the contribution of CIN to the molecular mechanisms governing relatively early changes in tumor progression remains to be fully understood [25, 26], especially in an *in vivo* context. In view of recent findings that cyclin D1 is capable of inducing aneuploidy and prior findings that the cyclin D1 kinase function appears to be dispensable for several activities, and because of the crucial implications of this mechanism for cancer therapeutics, we determined the importance of cyclin D1 kinase function in the induction of CIN and mammary tumorigenesis *in vivo*.

## RESULTS

### Cyclin D1 induction of mitotic abnormalities is kinase-independent

Recent studies using SKY analysis and gene expression profiling have demonstrated that re-expression of *cyclin D1^WT^* in cyclin D1-deficient cells results in CIN [22]. In order to test the kinase-independent function of cyclin D1 in aneuploidy and tumorigenesis, we utilized a cyclin D1 point mutant, cyclin D1 K112E (cyclin D1^KE^), which contains a lysine to glutamine substitution at amino acid position 112 (Supplementary Figure S1A). The cyclin D1^KE^ mutant was unable to bind CDKs *in vitro* [27]. Cyclin D1^KE^ immunoprecipitated CDK4 and CDK6, and could efficiently bind p27^Kip1^, however in an *in vitro* kinase assay the cyclin D1^KE^ complex showed dramatically reduced phosphorylation of pRb [28]. Cyclin D1^KE^ *in vivo* binds CDK4 and p27^Kip1^ however the phosphorylation of pRb *in vivo* was reduced similar to levels seen in *cyclin D1^−/−^* mice [29]. In MEFs cyclin D1^KE^ failed to bind CDK4 or p27^Kip1^ [16]. Collectively these studies demonstrate that the kinase function of cyclin D1^KE^ is abrogated or substantially blunted.

**Figure 1:**
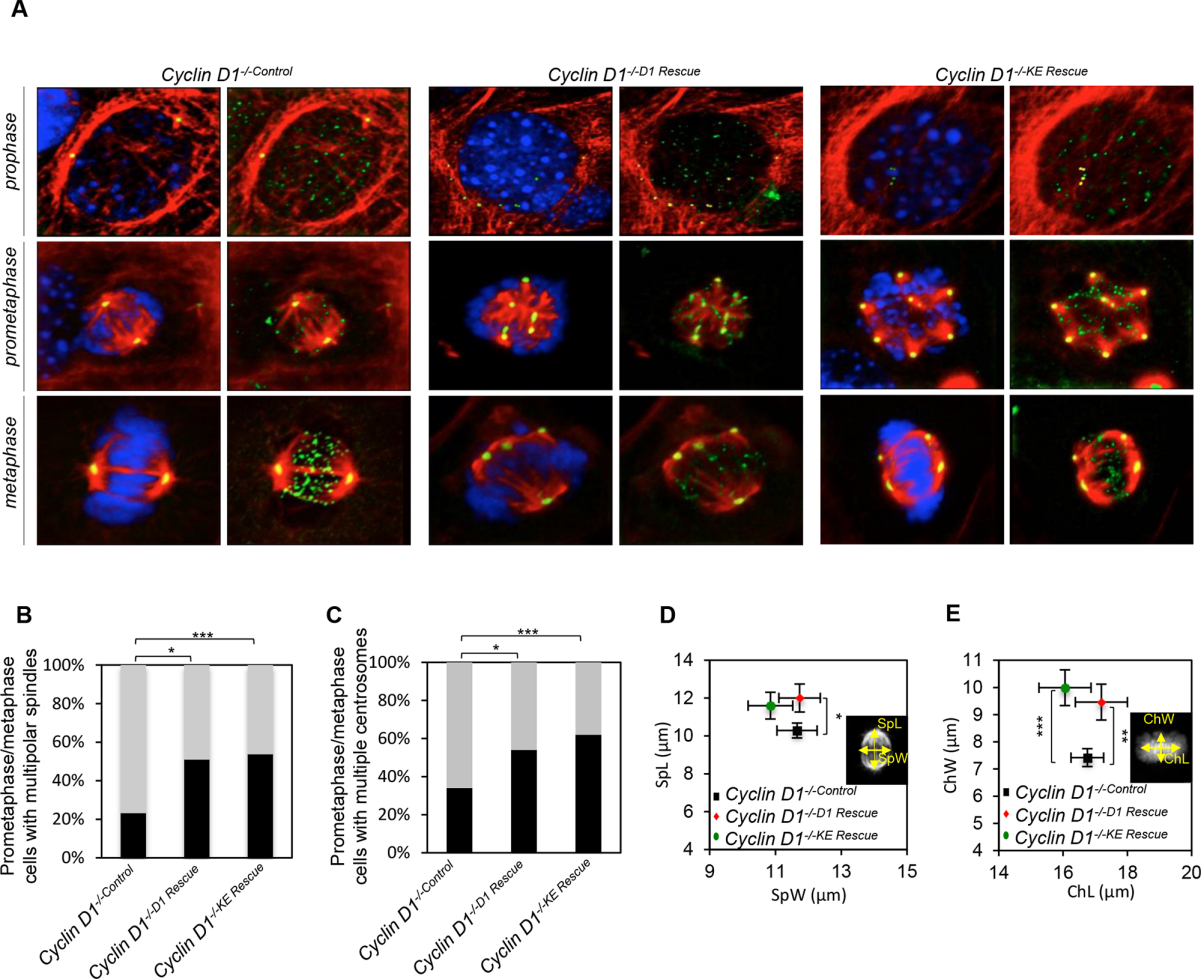
Cyclin D1 induction of centrosome amplification and mitotic spindle disorganization is independent of cyclin D1 kinase activity **(A)** Representative confocal maximum Z projections of mitotic cells from *cyclin D1^−/−Control^*, *cyclin D1^−/−D1 Rescue^* and *cyclin D1^−/−KE Rescue^*. Cells were immunostained for α-tubulin (red), γ-tubulin (yellow), crest (green), and Hoechst (blue). Scalebar 5 μm. **(B)** Frequencies of mitotic cells with multiple polar spindles (**p = 0.0051, ***p = 0.0004; calculated by Fisher contingency test). **(C)** Frequency of cells with multiple chromosomes (*p = 0.021, ***p = 0.0007; calculated by Fisher contingency test). **(D and E)** Spindle measurements on maximum Z projections of metaphase *cyclin D1^−/−^*, *cyclin D1^−/−D1 Rescue^* and *cyclin D1^−/−KE Rescue^* cells. Measurement of metaphase plate dimensions (DAPI): ChL, chromatin length; ChW, chromatin width (**p = 0.0087, ***p < 0.001). Measurement of spindle dimensions (tubulin): SpW, spindle width; SpL, spindle length (*p = 0.0486; data are mean of ± SEM).

Prior to engaging in studies to question whether the induction of aneuploidy by cyclin D1 is kinase-independent we verified the relative abundance and nuclear localization of cyclin D1^KE^. In *cyclin D1^−/−^* cells rescued with either *cyclin D1^WT^* or *cyclin D1^KE^*, the protein abundance was similar between the two cell lines (Supplementary Figure S1B). In addition there was no difference in the abundance within the nuclear compartment (Supplementary Figures S1C and S1D). Next we, determined the subcellular compartmentalization of cyclin D1^KE^ and cyclin D1^WT^. We compared 3T3 wild type cells to 3T3 wild type cells transduced with MSCV-*Cyclin D1^KE^* and the localization of exogenous cyclin D1^KE^ and endogenous cyclin D1^WT^ protein monitored during aphidocoline block in G1 to release into S phase. Cyclin D1^KE^, like endogenous cyclin D1^WT^, was exported from the nucleus to the cytoplasm (Supplementary Figure S2). Next, to determine whether the alterations in mitotic abnormalities were induced by cyclin D1^WT^ via its CDK-activating function, we performed immunofluorescence followed by high resolution confocal imaging of *cyclin D1^−/−^* 3T3 cells, rescued with either *cyclin D1^WT^* or *cyclin D1^KE^* (Figure 1A). The number of cells with multi-polar spindles was increased 28% in the *cyclin D1^−/−D1 Rescue^* cells and 31% in the *cyclin D1^−/−KE Rescue^* cells compared to control (*p* = 0.0051 and *p* = 0.0004 respectively) (Figures 1A and 1B). The generation of multi-polar spindle cells arising from abnormalities in centrosome number and distribution were quantitatively assessed using α-tubulin staining in conjunction with γ-tubulin. The *cyclin D1^−/−D1 Rescue^* and the *cyclin D1^−/−KE Rescue^* increased the percentage of prometaphase/metaphase cells with multiple centrosomes by 20% (*p* = 0.0021) and 28% (*p* = 0.0007) respectively compared to control cells (Figures 1A and 1C). The alteration of spindle architecture associated with metaphase plate disruption was measured by assessing metaphase plate length and width (ChL, Chw) and spindle length and width (SpL, SpW) (Figures 1D and 1E). Consistent with the increase in spindle/centrosome abnormalities, the ChW and SpL were significantly increased in *cyclin D1^−/−D1 Rescue^* and *cyclin D1^−/−KE Rescue^* cells compared with *cyclin D1^−/−Control^* cells.

### Cyclin D1^KE^ induces aneuploidy

**Figure 2:**
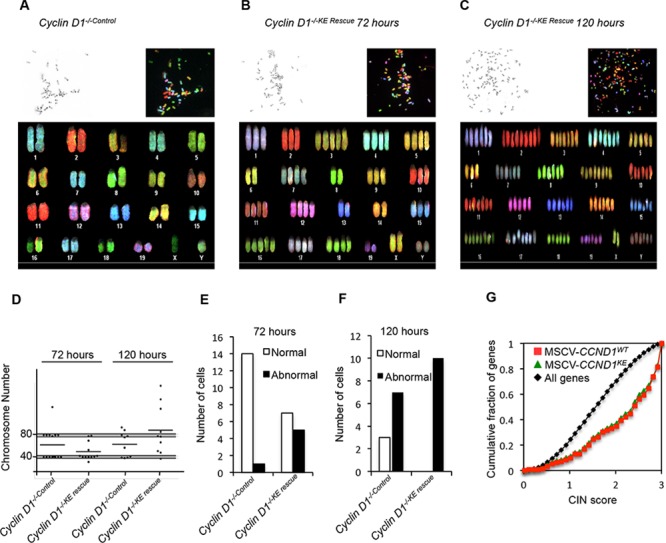
Cyclin D1 kinase-independent induction of aneuploidy Representative metaphases from spectral karyotyping (SKY) on MEFs of *cyclin D1^−/−Control^* at 72 hours (P6) **(A)**, *cyclin D1^−/−KE Rescue^* at 72 hours (P6) **(B)** and *cyclin D1^−/−KE Rescue^* at 120 hours **(C)**. Each panel contains the following images: inverted 4′,6-diamidino-2-phenylindole (DAPI) image of the metaphase (top left corner), raw spectral image of the metaphase (top right) and classified metaphase of the same metaphase (lower panel). **(D)** Scatter plots of chromosomal number across metaphase spreads from *cyclin D1^−/−Control^* and *cyclin D1^−/−KE Rescue^* cells showing the total number of chromosomes at 72 hours and 120 hours from cells with the noted genotype. The grey shaded bar represents expected deviation from normal at 2N and 4N (+/– 2 chromosomes). Applying the chi-square test of association by comparing *cyclin D1^−/−^* versus the *cyclin D1^−/−D1 Rescue^* MEFs, and *cyclin D1^−/−KE Rescue^* cells yields *p* < 0.001. **(E and F)** Bar graphs showing the number of normal and abnormal karyotypes comparing *cyclin D1^−/−Control^* and *cyclin D1^−/−KE Rescue^* at 72 hours and 120 hours post transduction. **(G)** An expression profile for *cyclin D1^−/−D1 Rescue^* (red line) and *cyclin D1^−/−KE Rescue^* (green line) induced genes [16] enriched for high CIN score (*p* < 0.0001).

Spectral karyotyping (SKY) was conducted comparing *cyclin D1^−/−KE Rescue^* vs. *cyclin D1^−/−Control^* cells. Aneuploidy refers to the loss or gain of whole or partial chromosomes resulting in a complement that differs from an exact multiple of the haploid number. In order to assess the role of cyclin D1^KE^ in aneuploidy we performed SKY analysis at 72 hours and 120 hours after rescue of *cyclin D1^−/−^* MEFs. Representative metaphase spreads are shown from analysis of all metaphases (Figures 2A–2C and Supplementary Figure S3A–S3C). At 72 hours *cyclin D1^−/−KE Rescue^* induced aneuploidy in 42% of cells, compared to 7% in *cyclin D1^−/−Control^* cells. At 120 hours, 100% of *cyclin D1^−/−KE Rescue^* cells demonstrated aneuploidy compared to 70% in *cyclin D1^−/−Control^* MEFs (Figure 2D, and 2F). Therefore, induction of aneuploidy by cyclin D1 is kinase-independent. SKY analysis assigns chromosomal rearrangements classified as deletions, duplications and translocations. There was no significant difference in chromosomal rearrangements between *cyclin D1^−/−Control^* and *cyclin D1^−/−KE Rescue^* MEFs.

To further assess the role of cyclin D1 kinase activity in aneuploidy induction we transduced MEFs with cyclin D1^KE^ in the presence and absence of a CDK4/6 antagonist, PD0332991, and assessed the induction of aneuploidy. Karyotyping was conducted comparing *cyclin D1^−/−KE Rescue^* vs *cyclin D1^−/−Control^* cells. Western blot analysis confirmed CDK4/6 antagonist PD0332991 diminished phosphorylation of pRB at S780 in *cyclin D1^−/−Control^* and *cyclin D1^−/−KE Rescue^* MEFs; *cyclin D1^−/−D1 Rescue^* 3T3 cells were used as a positive control for induction of phosphorylation of pRB at S780 (Supplementary Figure S4A). Representative metaphase spreads and numerical quantitation are shown from analysis of all metaphases from PD0332991 and vehicle treated MEFs (Supplementary Figure S4B). At 72 hours in presence of vehicle *cyclin D1^−/−KE Rescue^* induced aneuploidy in 67% (*p* = 0.027) of MEF cells, compared to 33% in *cyclin D1^−/−Control^* cells (Supplementary Figure S4C and S4D). At 72 hours in presence of PD0332991, *cyclin D1^−/−KE Rescue^* induced aneuploidy in 87% of cells (*p* < 0.001), compared to 20% in *cyclin D1^−/−Control^* cells (Supplementary Figure S4C and S4E).

In addition to using a CDK4/6 antagonist we also investigated the induction of aneuploidy by cyclin D1 in *cdk4/6^−/−^* 3T3 cells. *Cdk4/6^−/−^* 3T3 cells were transduced with cyclin D1^WT^ and cyclin D1^KE^ and we assessed the induction of aneuploidy. Western blot analysis of the cell lysates confirmed the cells were *cdk4^−/−^* and expressed exogenous cyclin D1^WT^ and cyclin D1^KE^ (Supplementary Figure S5A). At 72 hours in *cdk4/6^−/−^* 3T3 cells cyclin D1^WT^ and cyclin D1^KE^ induced aneuploidy in 67% of cells (*p* = 0.045) and 83% of cells respectively (*p* = 0.002), compared to 44% in *cdk4/6^−/−Control^* cells (Supplementary Figure S5B, S5C, and S5D). Therefore induction of aneuploidy by cyclin D1^WT^ and cyclin D1^KE^ is CDK independent.

Analysis of microarray data of *cyclin D1^−/−KE Rescue^* and *cyclin D1^−/−D1 Rescue^* vs. *cyclin D1^−/−Control^* MEFs demonstrated increased expression of genes associated with a high CIN score [30]. The CIN score was derived by a computational approach to define a gene expression signature that correlates with functional aneuploidy in tumors. The signature predicted poor outcome in 12 cancer data sets from six cancer types. The higher CIN score genes regulate the DNA damage checkpoint, spindle checkpoint and spindle assembly. The induction of high CIN score genes by cyclin D1 was independent of its kinase function (Figure 2G).

### Acute induction of Cyclin D1^KE^ leads to expression of high CIN score genes *in vivo*

**Figure 3:**
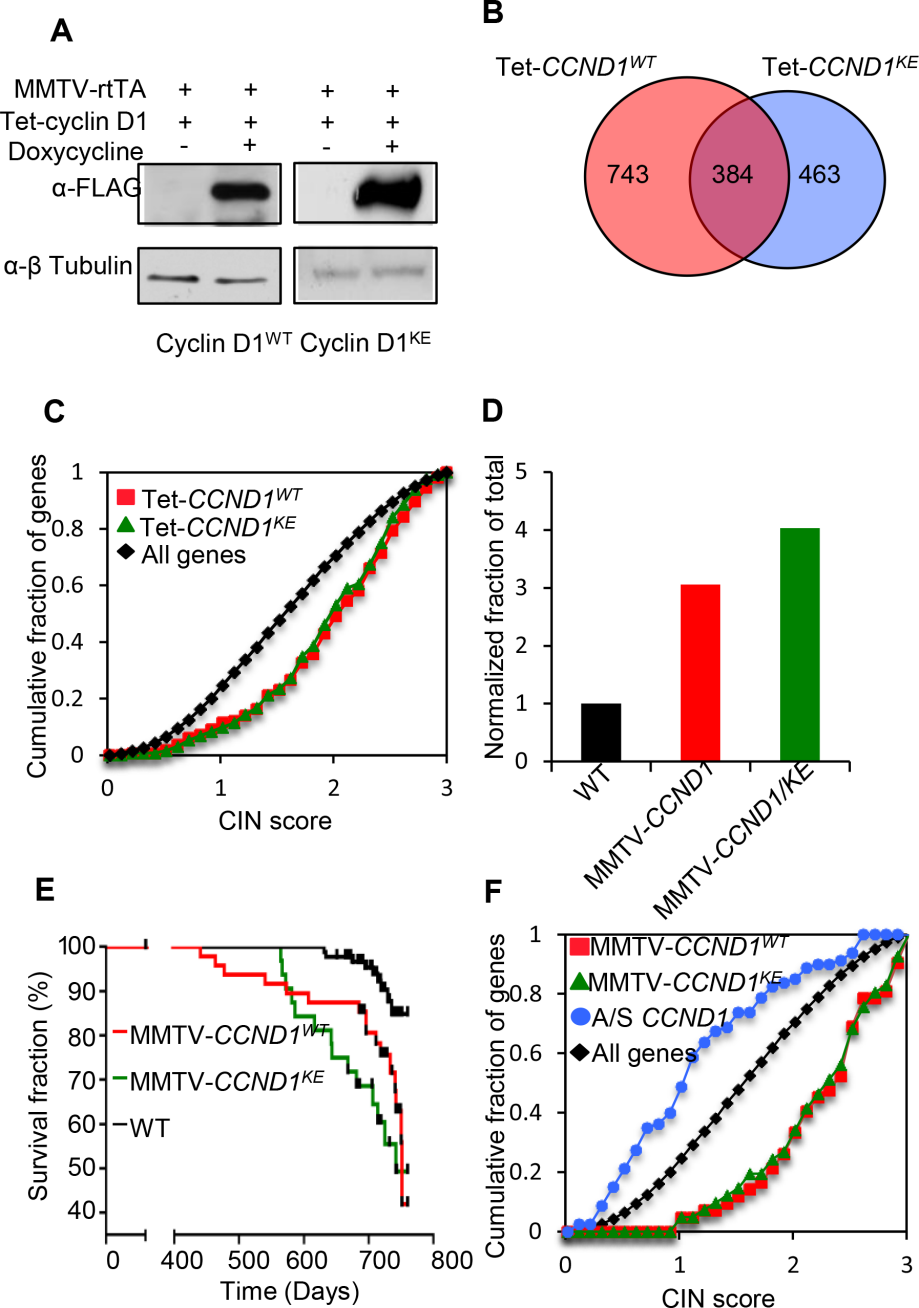
Cyclin D1 induces CIN genes *in vivo* and mouse mammary tumorigenesis independent of its kinase activity **(A)** Western blot using anti-FLAG of mammary gland protein lysates from Tet-*CCND1^WT^* and Tet-*CCND1^KE^* mice treated with doxycycline compared to control (Left panel). **(B)** Venn diagram representing genes differentially regulated by Tet-*CCND1^WT^* (*n* = 3) and Tet-*CCND1^KE^* (*n* = 3) (Right panel). 1-way ANOVA was used to evaluate the significance of differential expression between biological conditions. Data represents *p* < 0.05 and fold change in gene expression >1.5. **(C)** The most highly differentially regulated genes (Fold >2, *p* < 0.05) for Tet-*CCND1^WT^* (red line) and Tet-*CCND1^KE^* (green line) induced genes [16] are enriched for high CIN score (*p* < 0.0001). **(D)** Tumor incidence was markedly increased in MMTV*-CCND1^WT^* mice and MMTV*-CCND1^KE^* mice compared to WT mice. **(E)** Kaplan–Meier survival curves from mammary tumors of MMTV*-CCND1^WT^* (red line) and MMTV*-CCND1^KE^* (green line). **(F)** The most highly differentially regulated genes (Fold >2, *B* >3) for MMTV*-CCND1^WT^* (red line) and MMTV*-CCND1^KE^* (green line) induced genes are enriched for high CIN score (*p* < 0.0001). mRNA from the mammary glands of ponasterone A inducible cyclin D1 antisense mice [12] were subjected to microarray analysis demonstrated reduced CIN gene expression for cyclin D1 induced genes (*p* < 0.0001).

To directly determine the role of cyclin D1-mediated kinase activity in promoting mammary tumorigenesis, transgenic mouse models were deployed using either the tetracycline-inducible cyclin D1 transgenic mice (*rtTA/CCND1*), the Ponasterone inducible mammary epithelial cell targeted cyclin D1-antisense or the MMTV-cyclin D1 transgenic mouse model [12, 22] (Supplementary Figures S6A and S6C). Mammary-targeted expression of cyclin D1 was achieved by crossing transgenic mice carrying a mammary gland targeted recombinant Tetracycline transcription factor (*rtTA*-Tet ON system) to transgenic mice bearing an *rtTA*-responsive promoter driving either *cyclin D1^WT^* or *cyclin D1^KE^* (P_Tet_-CCND1^WT^ and P_Tet_-CCND1^KE^). The resulting offspring double positive for the transgenes were designated *rtTA*/CCND1^WT^ and *rtTA/*CCND1^KE^ (Supplementary Figure S6B). Pregnant females (14 days post coitus) were treated with tetracycline for 7 days, followed by sacrifice of the animals and removal of the thoracic mammary glands for further studies. Western blot analysis verified the induction of the cyclin D1 transgene (Figure 3A). Microarray analysis for gene expression profiles of the mammary glands identified gene clusters regulated by cyclin D1^WT^ and cyclin D1^KE^ (Figure 3B, Supplementary Figure S7A and Dataset S1). There was significant overlap between the gene expression profile regulated by cyclin D1^WT^ and cyclin D1^KE^ (*p* < 1 × 10^−10^). Pathway analysis of the genes in common between rtTA/CCND1^WT^ and rtTA/CCND1^KE^ revealed many functional terms previously identified as being cyclin D1 regulated including cell cycle and mitosis (Supplementary Figure S7B). Notably, the rtTA/CCND1^WT^ gene profile was enriched for high CIN score genes to a similar level as the rtTA/*CCND1^KE^* gene profile (Figure 3C). Therefore, acute expression of cyclin D1^KE^ was sufficient to induce CIN gene expression profiles within 7 days.

### Sustained mammary gland expression of cyclin D1^KE^ induces tumors independent of kinase

Next we employed mammary gland targeted cyclin D1 for a sustained expression study (Supplementary Figure S6C). MMTV-cyclin D1^KE^ and MMTV-cyclin D1^WT^ transgenic mice were monitored twice weekly for the development of mammary tumors. All mice in the tumor kinetics study were nulliparous, thus eliminating any potentially confounding effects of parity on tumor development in the FVB strain. Mice that developed palpable tumors were sacrificed within a week of tumor detection. MMTV-cyclin D1^KE^ tumor incidence (43.8%; *n* = 32 mice) was similar to MMTV-cyclin D1^WT^ (33.3%; *n* = 48 mice) (*p* = 0.358) with a 4-fold (*p* = 0.0002) and 3-fold (*p* = 0.0002) greater incidence, respectively, compared to the wild type mice (*n* = 92 mice) (Figure 3D). A Kaplan–Meier survival (Mammary gland tumor free survival) plot and analyses with a logrank test for curve comparisons were performed among all three lines and between paired lines. The event plotted was the date of sacrifice of the mice that developed tumors. Mice were censored on the date at which they were no longer followed. This included, 1) those that died unrelated to tumor prior to 760 days (censored on the date of death) and 2) those alive without tumor at the end of the study (censored on day 760). Kaplan–Meier survival plots demonstrated kinetics that was similar for both MMTV-cyclin D1^KE^ and MMTV-cyclin D1^WT^ animals (logrank *p* = 0.237) but significantly different from wild type mice (*p* < 0.0001 and *p* = 0.0037, respectively) (Figure 3E). Next, we performed histological analysis of the tumors from MMTV-cyclin D1^WT^ and MMTV-cyclin D1^KE^ mice. The spectrum of histological subtypes of the mammary cancers was similar between MMTV-cyclin D1^WT^ and MMTV-cyclin D1^KE^ mice (Supplementary Table S1). Indeed, it's the same spectrum that is seen in the ‘background’ of mammary cancers developing spontaneously in wild type mice.

Protein abundance from MMTV-cyclin D1^KE^ transgene in the mammary tumors was similar to MMTV-cyclin D1^WT^ in the mammary gland (Supplementary Figure S8A). The phosphorylation status of a CDK4/6 target site in pRB was substantially reduced in mammary gland tumors of MMTV-cyclin D1^KE^ compared to MMTV-cyclin D1^WT^ tumors (Supplementary Figure S8B). Gene expression profiles of the mammary tumors for MMTV-cyclin D1^KE^ and MMTV-cyclin D1^WT^ mice showed highly significant overlap (*p* < 1 × 10^−10^) (Supplementary Figure S9A–S9C and Dataset S2). Furthermore, enrichment for CIN gene expression was observed with both MMTV-cyclin D1^WT^ and MMTV-cyclin D1^KE^ (Figure 3F). There were no significant differences in the CIN score between the MMTV-cyclin D1^WT^ and MMTV-cyclin D1^KE^ tumors. Conversely, mammary epithelial cells from transgenic mice with targeted cyclin D1 anti-sense induced by ponasterone [12] showed a reciprocal change in CIN gene expression (Figure 3F), highlighting a role for endogenous cyclin D1 in maintaining basal CIN gene expression. Reintroduction of either cyclin D1^WT^ or cyclin D1^KE^ into *cyclin D1^−/−^* MEFs, transient expression in the mammary gland in transgenic mice, or sustained expression under control of the MMTV promoter, was sufficient for the induction of CIN gene expression; therefore these functions of cyclin D1 are kinase-independent.

### Recruitment of cyclin D1 to local chromatin is kinase-independent

**Figure 4:**
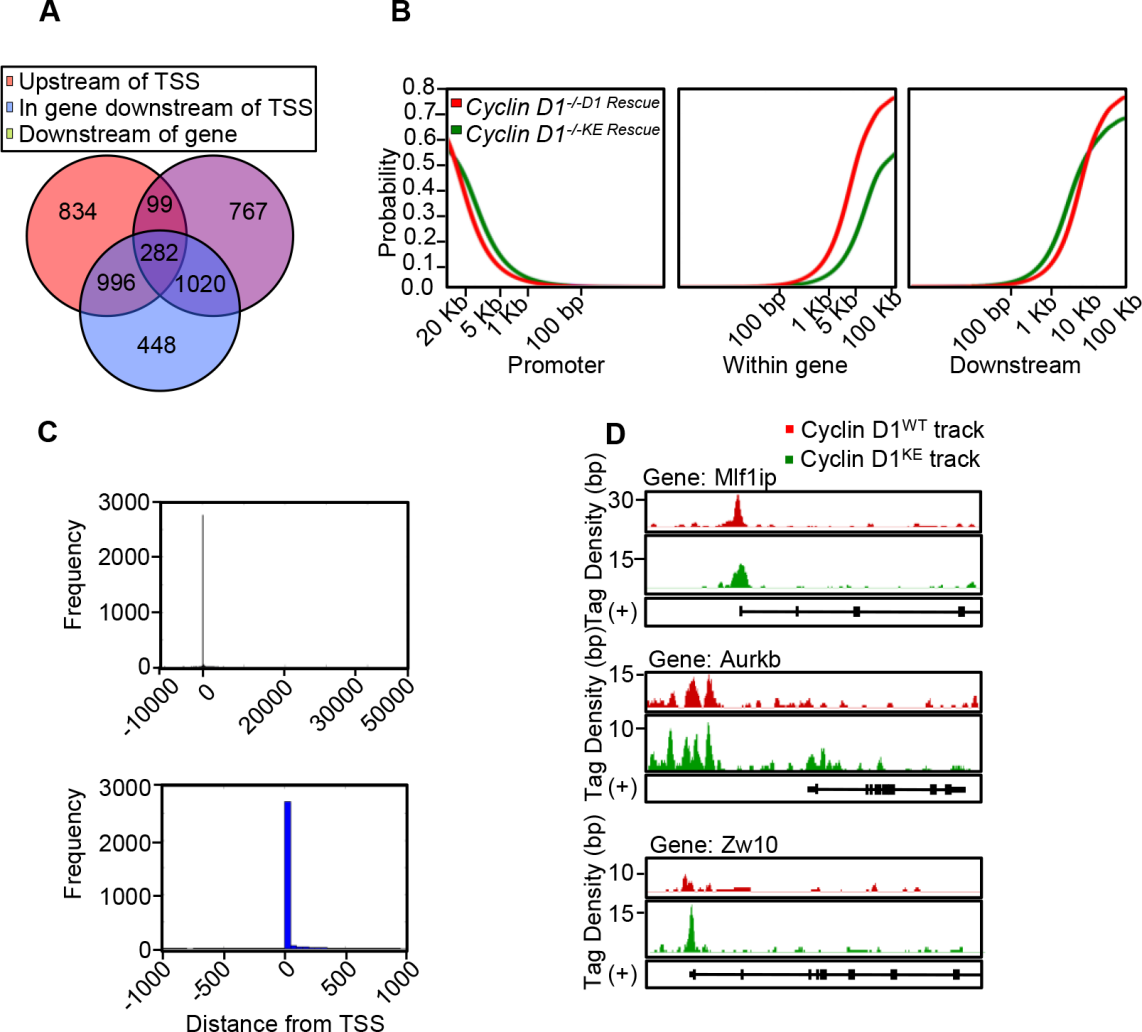
Chip-Seq demonstrates similar characteristics of genomic occupancy for cyclin D1^KE^ and cyclin D1^WT^ **(A)** Venn diagram showing distribution of the 4446 intervals with respect to neighboring genes. The interval is depicted in relation to transcriptional start site (TSS). Upstream of TSS defined as –10 kb to 0 kb. Downstream defined as 0 kb to +10 kb after transcriptional stop site. **(B)** The cumulative fraction of intervals from cyclin D1^WT^ and cyclin D1^KE^ mutant that are within the upstream, intergenic or downstream regions of a gene. **(C)** Histogram of cyclin D1 bound regions relative to transcriptional start point at –10 kb to +50 kb (Upper panel) and –1 kb to +1 kb (Lower panel). **(D)** Integrated genome browser visualization of tag density profiles for ChIP-Seq cyclin D1^WT^ and ChIP-Seq cyclin D1^KE^. Selected genes are, MLF1 interacting protein (*Mlf1ip*-a kinetochore platform protein), aurora kinase B (*AurkB*-member of chromosomal passenger complex) and zeste white 10 homolog (*Zw10*-mitotic check point protein).

Cyclin D1 regulates transcription factor (TF) occupancy in chromatin and a cyclin D1-DNA bound form occupies promoter-regulatory regions in the context of local chromatin [21, 23]. In order to determine whether DNA association in chromatin was kinase-dependent we conducted genome wide analysis comparing the cyclin D1^WT^ and cyclin D1^KE^ mutant using ChIP-Seq analysis. The distribution of binding sites by ChIP-Seq in relation to the transcriptional start sites demonstrated binding of active regions within the promoter-region and beyond 10 kb, consistent with a model in which cyclin D1 localizes to both very distal elements and promoter proximal regulatory elements (Figure 4A and 4B) (Supplementary Table S2 and Dataset S3). The tag density profiles for cyclin D1^WT^ and cyclin D1^KE^ demonstrated a similar distribution of genomic association when comparing location at the promoter, within a gene or downstream of the transcriptional start site (Figure 4B). In addition, as in cyclin D1^WT^, the tag density profiles for cyclin D1^KE^ were enriched at the transcriptional start sites (Figure 4C). Chip-Seq analysis demonstrated significant overlap between cyclin D1^WT^ and cyclin D1^KE^ gene occupancy (1068 genes in common, *p* = 4.48 × 10^−11^). Comparison to a previously published gene set from cyclin D1 associated genes by ChIP-ChIP also showed a significant overlap (1505 intervals in common, *p* = 0.0018, 1144 genes in common, *p* = 1.61 × 10^−12^ [21]).

**Figure 5:**
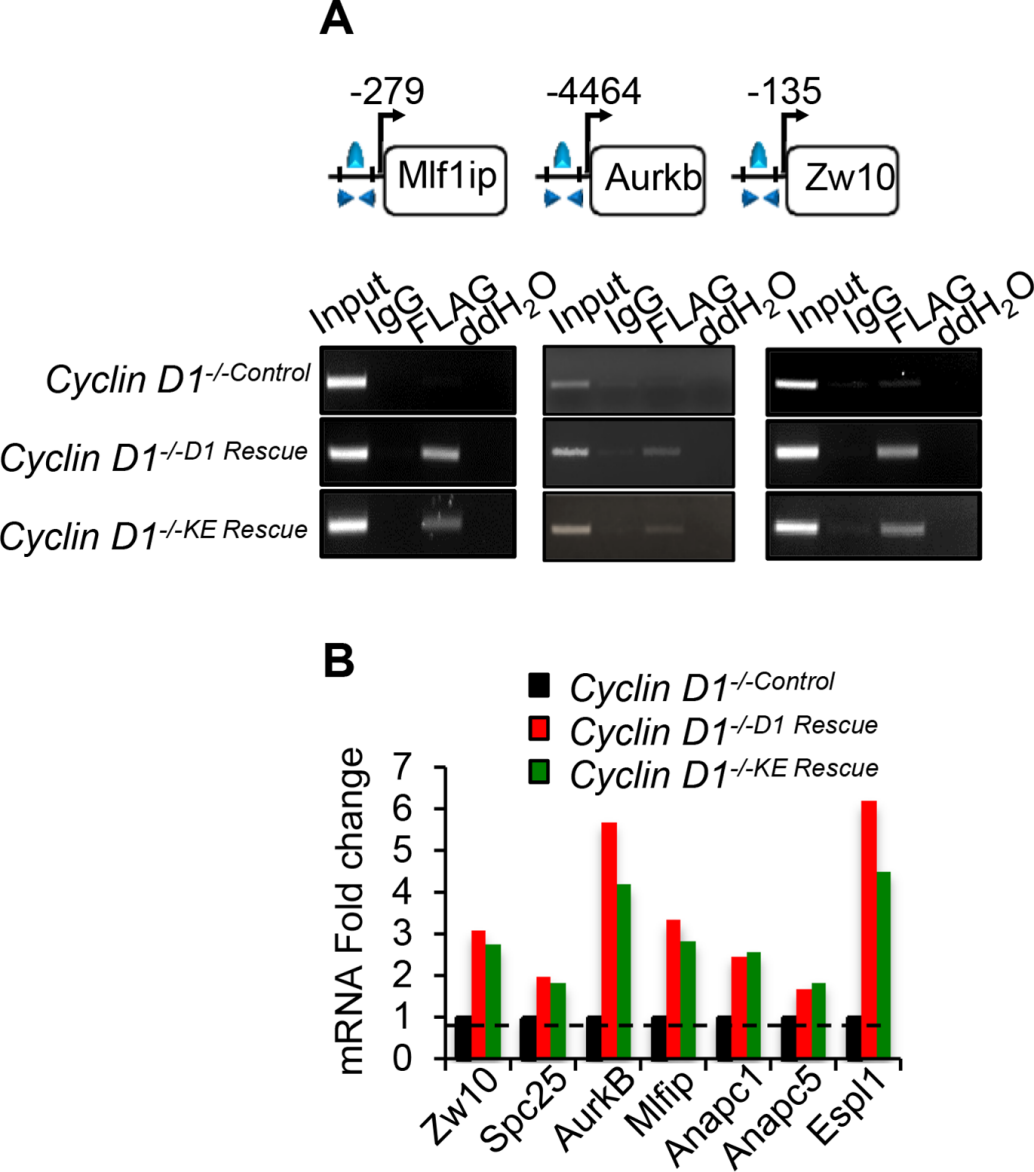
Cyclin D1^WT^ and cyclin D1^KE^ associate with and promotes expression of genes involved in mitosis **(A)** Chromatin immunoprecipitation (ChIP) assay performed to assess the association of cyclin D1^WT^ and cyclin D1^KE^ mutant on the promoter regions of selected genes. **(B)** Quantitative PCR on target mRNAs selected based on cyclin D1^KE^ associated genes. Normalized expression ratio of *cyclin D1^−/−^* cells with MSCV-FLAG/*CCND1* compared to MSCV-control.

**Figure 6:**
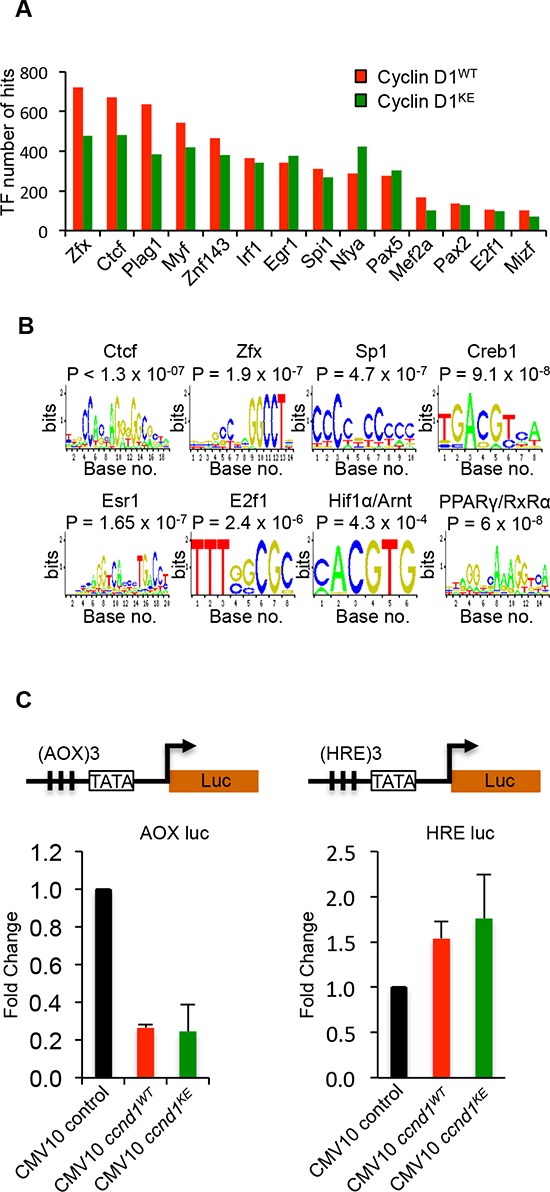
Identification of transcription factor motifs found in cyclin D1^WT^ and cyclin D1^KE^ interval sequences **(A)** Selection of transcription factor motif hits common between Cyclin D1^WT^ and cyclin D1^KE^ interval sequences **(B)** Representative TF motifs found in the interval regions associated with cyclin D1^WT^ and cyclin D1^KE^ **(C)** Luciferase reporter gene assays were conducted using the Peroxisome Proliferator-Activated Receptor γ (AOX-LUC) (left panel) and Hypoxia Responsive Element (HRE-LUC) (right panel) luciferase reporter constructs. The number of responsive elements for each construct is depicted in the reporter schematic. HEK293T cells were co-transfected with cyclin D1 (50 ng). Data are of *n* = 2 separate experiments, mean ± SEM.

Select CIN associated genes showed similar ChIP-Seq tag density profiles for cyclin D1^WT^ and cyclin D1^KE^ (Figure 4D). ChIP analysis of selected target genes governing CIN demonstrated similar relative occupancy for cyclin D1^WT^ and cyclin D1^KE^ (Figure 5A). We then analyzed a broader array of genes governing CIN by QT-PCR, demonstrating similar upregulation of the transcript level by cyclin D1^WT^ and cyclin D1^KE^ (Figure 5B). The enrichment for transcription factor (TF) binding sites identified TF motifs and their statistical significance for the cyclin D1^WT^ and cyclin D1^KE^ (Supplementary Figure 6A and Supplementary Table S3). For the examples shown the prevalence of the TF binding site was similar and significant for both cyclin D1^WT^ and cyclin D1^KE^. Representative TF motifs most significantly enriched in the cyclin D1^WT^ intervals are shown for the cyclin D1^KE^ intervals (Figure 6B). In addition to associating with the TF motifs, we verified that cyclin D1^KE^ regulated the reporter activity of selected TF responsive elements in a similar manner to cyclin D1^WT^ (Figure 6C).

## DISCUSSION

The current studies demonstrate that transient cyclin D1 overexpression induces CIN gene expression both in fibroblasts and in the mammary gland *in vivo*. Previous studies had carefully characterized a mutant of cyclin D1 (cyclin D1^KE^) demonstrating that it has substantially decreased cyclin-dependent kinase activity using pRb as a substrate [28, 29]. In the current studies, the cyclin D1^KE^ mutant was used to either rescue *cyclin D1^−/−^* MEF or was expressed in the mammary gland of transgenic mice. Either reintroduction of cyclin D1^KE^ into *cyclin D1^−/−^* MEF, transient expression in the mammary gland in transgenic mice, or sustained expression under control of the MMTV promoter, was sufficient for the induction of CIN gene expression. The induction of CIN gene expression by cyclin D1^KE^ was indistinguishable from the induction of CIN gene expression by cyclin D1^WT^.

D-type cyclins have been shown to physically bind and to either activate or repress activity of transcription factors [32–34]. In reporter gene assays this function was independent of the CDK activating function [34]. *In vivo* using *cyclin D1^−/−^* mice, the abundance of cyclin D1 was shown to be limiting in the recruitment of transcription factors in the context of local chromatin using ChIP assays [19]. ChIP identified cyclin D1 at transcription factor binding sites of endogenous gene promoters, associated with the recruitment of SUV39, HP1α, HDAC 1, 2, and p300 [20, 31]. Cyclin D1 determined the local acetylation and both di- and tri-methylation of histones [31]. Using serial ChIP analysis of non-coding miRNA regulatory regions, cyclin D1 was identified at the regulatory region of miR17/20 [35]. The current studies are consistent with a role for a DNA bound form of cyclin D1 governing gene expression independent of its kinase function. Furthermore these studies show through quantitative ChIP-Seq studies similar binding patterns for cyclin D1 independent of its kinase function to similar regions of the genome, and similar levels of binding to individual genes governing CIN.

In the current studies sustained expression of either cyclin D1^WT^ or cyclin D1^KE^ induced mammary tumors in transgenic mice with similar kinetics. Consistent with this experimental evidence for cdk independence of cyclin D1's role as a driver oncogene, human breast cancers overexpressing cyclin D1 do not show high levels of the canonical E2F target gene cyclin E [4, 5] and exhibit relatively normal proliferation rates compared to tumors with genetic deletion of pRb [4–6, 36]. Furthermore, cyclin D1 levels in tumors do not correlate with the marker of proliferating cells, Ki67 [36, 37]. The current studies demonstrate that forced expression of either cyclin D1^WT^ or cyclin D1^KE^ give very similar expression patterns of downstream gene expression, and raise the intriguing possibility that cyclin D1 primarily contributes to oncogenesis through regulating a transcriptional program implicated in CIN.

In contrast, in certain model systems cyclin D1 serves as a mediator of mammary tumorigenesis induced by other oncogenes such as ErbB2, the role of cyclin D1 is CDK-dependent. For example, *CDK4*^−/−^ mice and cyclin D1^KE^ knock-in mice are resistant to ErbB2-induced mammary tumorigenesis [14, 29, 38]. Together, these studies may illustrate two distinct scenarios reflecting two distinct clinical-pathological settings. Cyclin D1 is overexpressed in the majority of human breast tumors, many of these representing downstream effects through induction of cyclin D1 by oncogenic signals (Ras, MAPK [39]), Src [40], ErbB2 [41], STATs [42], Notch [43], NFκB [44]. Such tumors rely on kinase activity of cyclin D1, and tumor growth could be abrogated not only by inactivation of cyclin D1 but also by CDK4/6 inhibition. CDK4/6 inhibitors (Palbociclib, LEE011, LY2835219), currently in various stages of phase clinical trial, are showing promise as potential therapies in a range of human malignancies [45].

In contrast, cyclin D1 is often overexpressed as a function of genomic rearrangement or amplification. In this setting cyclin D1 is a primary driver oncogene and is experimentally recapitulated by targeted cyclin D1 transgene overexpression. Thus, the present evidence for a CDK-independent role of cyclin D1 in driving mammary tumorigenesis may be especially relevant to human breast cancer, particularly the large subset with clonally selected *cyclin D1* gene amplification and potentially the multiple other types of human tumors similarly driven by cyclin D1 amplification or rearrangement [1]. Accordingly, for these tumors, direct therapeutic targeting of cyclin D1 would be predicted to have more efficacy than CDK inhibitors.

## MATERIALS AND METHODS

### Cell culture and cell lines

The MSCV-IRES-GFP retroviral vector and cyclin D1 wild-type constructs were previously described [46]. *Cyclin D1^+/+^* and *cyclin D1^−/−^* primary MEF cultures were prepared as described previously. Cells were maintained in DMEM supplemented with 10% fetal bovine serum, 100 μg/ml each of penicillin and streptomycin. *Cdk4/6^−/−^* 3T3 cells were a gift from Dr. M. Barbacid.

### Generation of transgenic mice

Two 8 amino acid FLAG tagged constructs were prepared using either human cyclin D1 cDNA (pPL-8) [2] or an otherwise identical cyclin D1 cDNA bearing the “KE” mutation - an AAG to GAG that changes K (lysine) to E (glutamic acid) at amino acid 112, blocking cyclin D1 associated kinase activity. These constructs were inserted into the previously described MMTV-Sv40-BSSk vector [3] (see Supplementary Figure 1A and 5C) and its SalI—SpeI linearized fragment which included MMTV-LTR, the FLAG tagged construct, plus SV40 intron and polyadenylation signals, was microinjected into fertilized FVB/N mouse oocytes and implanted into pseudopregnant FVB fosters using standard methods. Pups were examined for successful insertion of the respective transgenes using tail genomic DNA and PCR primers for the SV40 cassette with confirmation by Southern blotting as described [3]. From these founders and progeny, two independent lines, called MFD1 and MFD1-KE, with robust and comparable levels of transgene expression in mammary tissue as determined by Northern blotting with a human cyclin D1 cDNA probe [1, 3], were selected for expansion and long-term analyses of tumor kinetics. The previously described MMTV-cyclin D1 (no FLAG tag) line MP1 [3] and FVB wild type (WT) mice were used as controls as indicated below.

The cDNA of human cyclin D1 including 3xFLAG sequence was amplified by PCR using p3xFLAG CMV 10-cyclin D1 as template. The restriction sites (Xho I/Not I) were introduced to the primers. The PCR fragment was cloned into pF43 vector. To prepare the DNA fragment for making transgenic mice, the pF43–3xFLAG-cyclin D1 vector was digested with Xho I/Not I/Pvu I. A 2.4-kb DNA fragment was recovered from agarose gel and purified for injection. Transgenic founder lines were backcrossed with wild type FVB mouse for three generations to obtain the stably inherited transgene line, followed by cross mating with MMTV-rtTA line (from Dr. Lewis Chodosh's lab) to obtain cyclin D1+/+ rtTA+/+ mice (Supplementary Figure 6A and 6B). 6–8 weeks-old female double transgenic mouse was used for further experiments. 8-week-old tetracycline-inducible cyclin D1/rtTA bi-transgenic pregnant female mice (12 days postcoitus) were administered doxycycline in the drinking water to a final concentration of  2 mg/ml. Following 7 days of doxycycline treatment, the mice were sacrificed and mammary glands extracted for tissue fixation and RNA/protein isolation.

### Retrovirus production and infection

Retroviral production and infection of *cyclin D1^−/−^* MEFs cells were described in detail previously [46].

### ChIP-Seq analysis and transcription factor enrichment

Detailed methods of chromatin preparation, labeling and construction of libraries have been documented previously [22]. For ChIP-Seq analysis, the 35-nt sequence reads (“tags”) identified by Illumina's Genome Analyzer 2 are mapped to the genome using the ELAND algorithm. Only tags that map uniquely, have no more than 2 mismatches, and that pass quality control filtering are used in the subsequent analysis. Since the 5′-ends of the sequence tags represent the end of ChIP/IP-fragments, the tags are extended in silico (Genpathway software) at their 3′-ends to a length of 110 bp, which is the average fragment length in the size selected library. To identify the density of fragments (extended tags) along the genome, the genome is divided into 32-nt bins and the number of fragments in each bin is determined. The ChIP-Seq peak intervals were determined using the MACs version 1.4 algorithm. We used the default values and provided the FLAG experiment with IgG as a background dataset. We used a *p*-value of 1.0E-5 as the cutoff for peak detection, which identified 4296 intervals. Supplementary Dataset 1 provides a further summary of the number of intervals found and their position relative to mouse genes. We used the DAVID Functional Annotation Tool to annotate functional enrichment. Transcription factor binding sites were computed as previously described [22].

In order to find the transcription factor binding sites we downloaded the latest version of the mouse genome, mm9, which was released in July 2007 from the USCS Main Genome Browser [47]. Using the Galaxy Toolbox [48] we extracted the sequence 10 kb upstream and downstream of each gene and submitted them to the Jasper server [49] with the default parameters to find all vertabrate transcription factor binding sites. We then assessed the overlap between these transcription factor binding sites and the cyclin D1^KE^ peak intervals. We used a permutation test initially proposed by Tuteja et al, [50]. In brief, this test involves creating psuedo-random in silico ChIP-Seq experiments that accurately reflects a null model of random binding. We shuffled the locations of the windows obtained from the cyclin D1^KE^ ChIP-Seq experiment and then counted the observed number of transcription factor binding sites. We calculate the *p*-value as the fraction of times in which the random count is larger then the observed count. For this experiment we performed 1.0E + 9 random permutations.

To determining overlap between cyclin D1^WT^ and cyclin D1^KE^ cyclin D1 binding-sites we used both a gene-based method and an interval based method. For the interval based method we used the same permutation method as described for the transcription factor enrichment to determine the overlap between the cyclin D1^WT^ and the cyclin D1^KE^ mutant binding sites. We used the intervals published previously for the cyclin D1^WT^ intervals [22]. Significance of overlap between cyclin D1^KE^ set and ChIP-ChIP data set [21] calculated using the same approach. For the gene-based method we used a hypergeometric test to determine the probability that cyclin D1^WT^ and cyclin D1^KE^ intervals are located in the promoter region or 2 kb upstream of the same genes.

In order to further examine the similarity of the enriched transcription factors we examined the number of transcription factor binding sites within the promoter region of each gene and in the cyclin D1^WT^ or cyclin D1^KE^ intervals. We then fit the difference in the counts between the corresponding transcription factors to a distribution using a Gaussian kernel density estimator [51]. Due to the discrete nature of the distribution we truncated to the maximum difference, 855 counts in this case, and re-normalized the distribution. We then calculated the *p*-value for each transcription factor as 1-cdf (delta).

### Chromatin immuno-precipitation assay (ChIP)

ChIP material was prepared in accordance with the Magna ChIP (Millipore) manufacturer's guidelines. Briefly, 3 × 10 cm plates of actively growing late passage MEFs cyclin D1^−/−^ MSCV-IRES-cyclin D1 were fixed for 10 min with paraformaldehyde 37% (final concentration 1%). Unreacted formaldehyde was quenched with 1 ml of 10 × glycine. The 3 plates were washed twice with ice cold PBS and the pellets harvested in 1 ml of PBS with protease inhibitor cocktail and pooled together in a 15 ml tube in order to obtain 1.5 × 10^6^ cells. DNA fragmentation of the pellets was achieved by sonication, 35 cycles of 20 seconds each at maximum speed using OMNI-Ruptor 4000 (OMNI International, Inc, Kennesaw, GA). Immuno-precipitation (IP) was performed with 10 μg of M2 FLAG antibody (Sigma-Aldrich, St. Louis, MO) and equivalent amount of mouse IgG as negative control. Washes and elution of the IP-DNA were performed according to the protocol. PCR primers were designed based on the peak interval sequence associated with cyclin D1 and the PCR products were visualized by agarose gel electrophoresis.

ChIP-DNA quantitation was conducted in an Agilent 2100 bio analyzer (Agilent Technologies, Palo Alto, CA), using Power SYBR Green (AB biosciences, Allston, MA) according to the manufacturer's guidelines. Equal quantities of ChIP-DNA were used for the real-time PCR quantitation. Ct values were used to calculate the relative fold enrichment (2-ΔCt, ΔCt = Ctinput–CtIgG). A one way ANOVA followed by Student's *t*-test comparison was performed to compare the relative fold enrichment (*n* = 3).

### Karyotype analysis

For SKY analysis, fluorescence color images of chromosomes stained by Rhodamine, Texas Red, Cy5, FITC and Cy5.5 were captured under a Nikon microscope equipped with a spectral cube and Interferometer module. SKY View software (version 1.62), was used to analyze chromosomal number and structural alterations of chromosomes, including simple balanced translocations, unbalanced (or nonreciprocal) translocations, deletions and duplications. At least 10 metaphases were analyzed per sample. Statistical significance calculated using chi-square test of association (Pearson).

### Real-time PCR

RNA quantitation was conducted in an Agilent 2100 bio analyzer (Agilent Technologies, Palo Alto, CA), using Power SYBR Green (AB biosciences, Allston, MA) according to the manufacturer's guidelines. Equal quantities of RNA were used for the reverse transcription reactions. Primers for all the genes were designed using GenScript's bioinformatics tools (GenScript, Piscataway, NJ).

### Microarray analysis

Genes with differential expression *p*-value ≤ 0.01 and absolute fold change ≥ 1.25). Mouse MG_U74Av2 microarrays were used for MSCV-rescued MEFs, Mouse 430A_2 microarrays were used for MMTV-Cyclin D1 model (GEO accession number—GSE43216). Arrays were processed as previously described [22]. CIN score enrichment was conducted as described, the comparison to CIN curves from Tet-*CCND1^WT^* and MMTV-*CCND1^WT^* has been published previously.

### Western blotting and luciferase assays

The following antibodies were used for Western blotting: Guanine Nucleotide Dissociation Inhibitor (GDI) [40], Cyclin D1 (NeoMarkers, MS-210-P), FLAG M2 antibody (Sigma Aldrich, #F1804), β-Tubulin (Sigma Aldrich, T4026), phosphorylated RB (S780) (Cell Signaling), cdk4 (H-22) (Santa Cruz Biotechnology Inc.). Luciferase assays were conducted as described previously described [52]. Assays were conducted using 50 ng of plasmid DNA and 100 ng reporter plasmid.

### Immunofluorescence and confocal analysis

Immunofluorescence was performed as described previously [22]. *Cyclin D1^−/−Control^*, *cyclin D1^−/−D1 Rescue^* and *cyclin D1^−/−KE Rescue^* subcellular localization was determined using the M2 anti-FLAG antibody (Sigma-Aldrich, #F1804). The whole cell fluorescence intensity per pixel^2^ was normalized to WT signal intensity. A one-way ANOVA followed by Student's *t*-test comparison was performed to compare percentage of fluorescence intensity for cyclin D1 abundance between *cyclin D1^−/−D1 Rescue^* and *cyclin D1^−/−KE Rescue^* cells (*n* = 20).

### Study approval

Animal studies were approved by the appropriate institutional animal care and oversight committees of the University of Connecticut and Thomas Jefferson University.

### Statistical analyses

MACs algorithm was employed to determine number of ChIP-Seq peaks (FDR = 4.35%). Analysis of transcription factor enrichment within the interval sequences produced by the ChIP-Seq data was computed using a permutation test. Enrichment for high CIN scoring genes [30] between two sets compared using Wilcoxon matched paired test. Kaplan–Meier plots were compared by log-rank test. For comparison between two independent groups, the Student's *t*-test was used (*p* < 0.05). Significance of karyotype analysis conducted using Chi-squared test of association.

## SUPPLEMENTARY FIGURES AND TABLES


