# Supplementary material for: Kinase-independent role of cyclin D1 in chromosomal instability and mammary tumorigenesis
Source: Oncotarget. 2015 Mar 25;6(11):8525–38. doi: 10.18632/oncotarget.3267 (PMC4496164; doi:10.18632/oncotarget.3267)
Supplement: Supplementary file 1 [file oncotarget-06-8525-s001.pdf]

SUPPLEMENTARY FIGURES AND TABLES

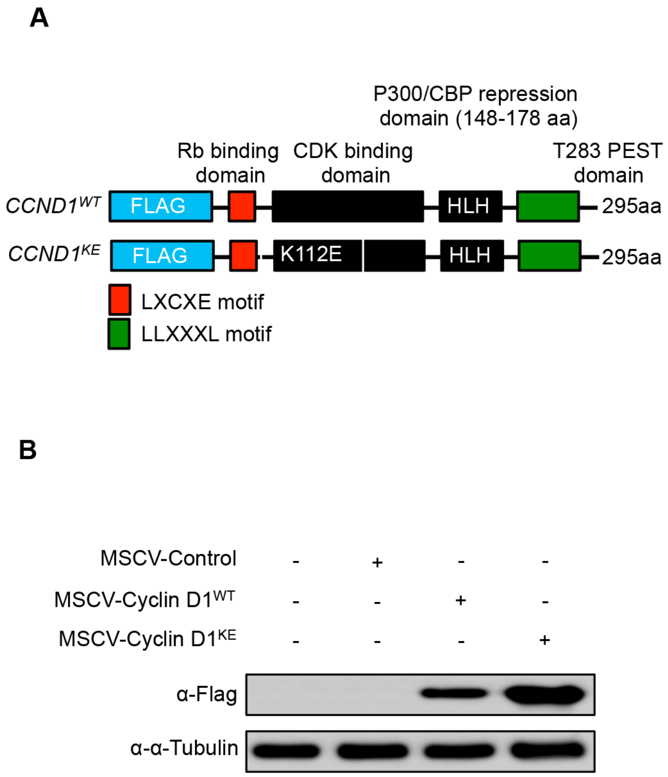

Supplementary Figure 1: Structure of Cyclin D1^{WT} and cyclin D1^{KE} vectors and comparison of their abundance in rescued 3T3 cells. (A) Schematic representation of Cyclin D1^{WT} and cyclin D1^{KE} proteins. **(B)** Western blot analysis on randomly cycling *cyclin D1*$^{-/-}$ and *cyclin D1*$^{-/-Control}$, *cyclin D1*$^{-/-D1\ Rescue}$ and *cyclin D1*$^{-/-KE\ Rescue}$ cells using anti-FLAG and anti-α Tubulin.

(*Continued*)

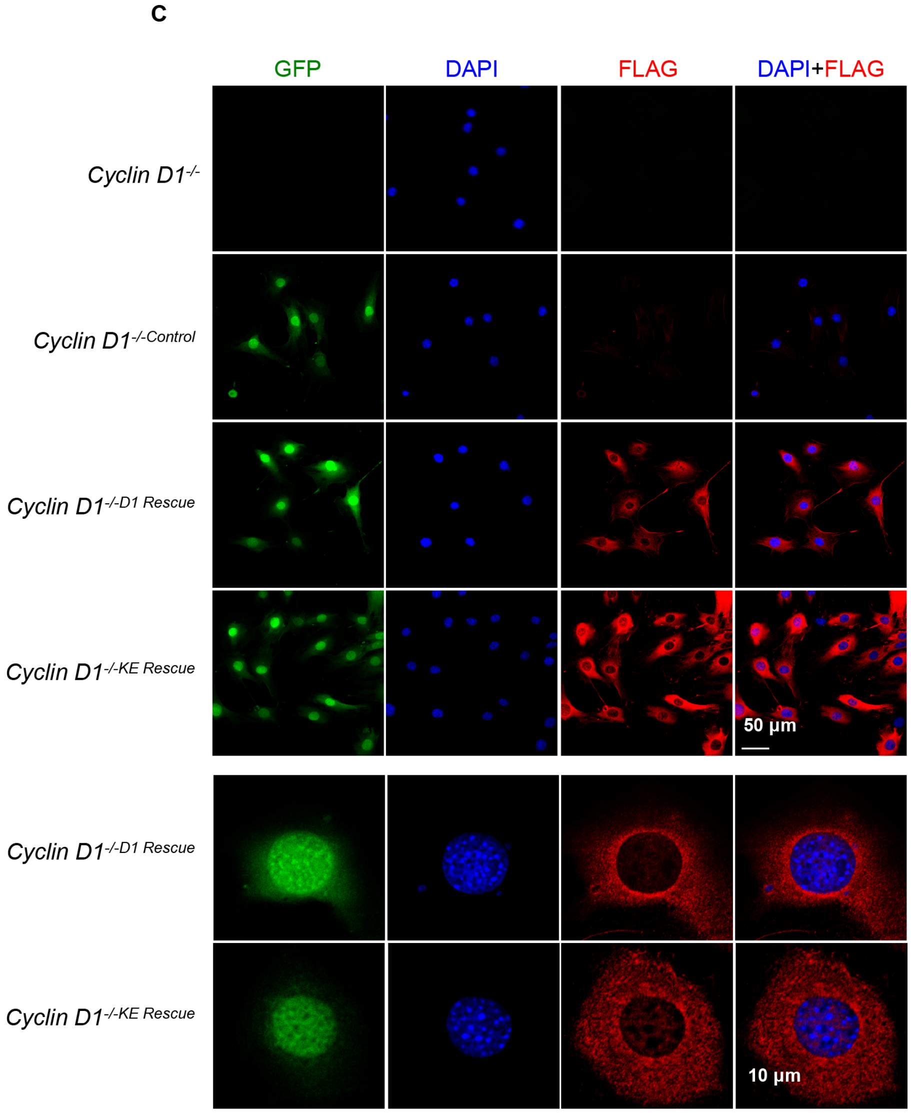

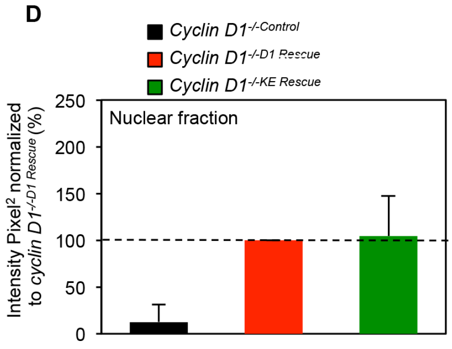

Supplementary Figure 1: (*Continued*) Structure of Cyclin D1^{WT} and cyclin D1^{KE} vectors and comparison of their abundance in rescued 3T3 cells. (C) Representative images of *cyclin D1$^{-/-Control}$*, *cyclin D1$^{-/-D1\ Rescue}$* or *cyclin D1$^{-/-KE\ Rescue}$* 3T3 fibroblast cells stained for DAPI (blue) and anti-FLAG (red) to the N-terminal FLAG-tag of cyclin D1 to discern subcellular distribution by IHC, scale bars 50 μm (upper panels) and 10 μm (lower panels). **(D)** Average fluorescent intensity of anti-FLAG signal in *cyclin D1$^{-/-Control}$*, *cyclin D1$^{-/-D1\ Rescue}$* *cyclin D1$^{-/-KE\ Rescue}$* 3T3 cells (*n* = 25 cells) within the nuclear compartment. Signal intensity from nuclear compartment normalized to cyclin D1^{WT}. No statistical difference was observed between cyclin D1^{WT} or cyclin D1^{KE} localization in the two lines.

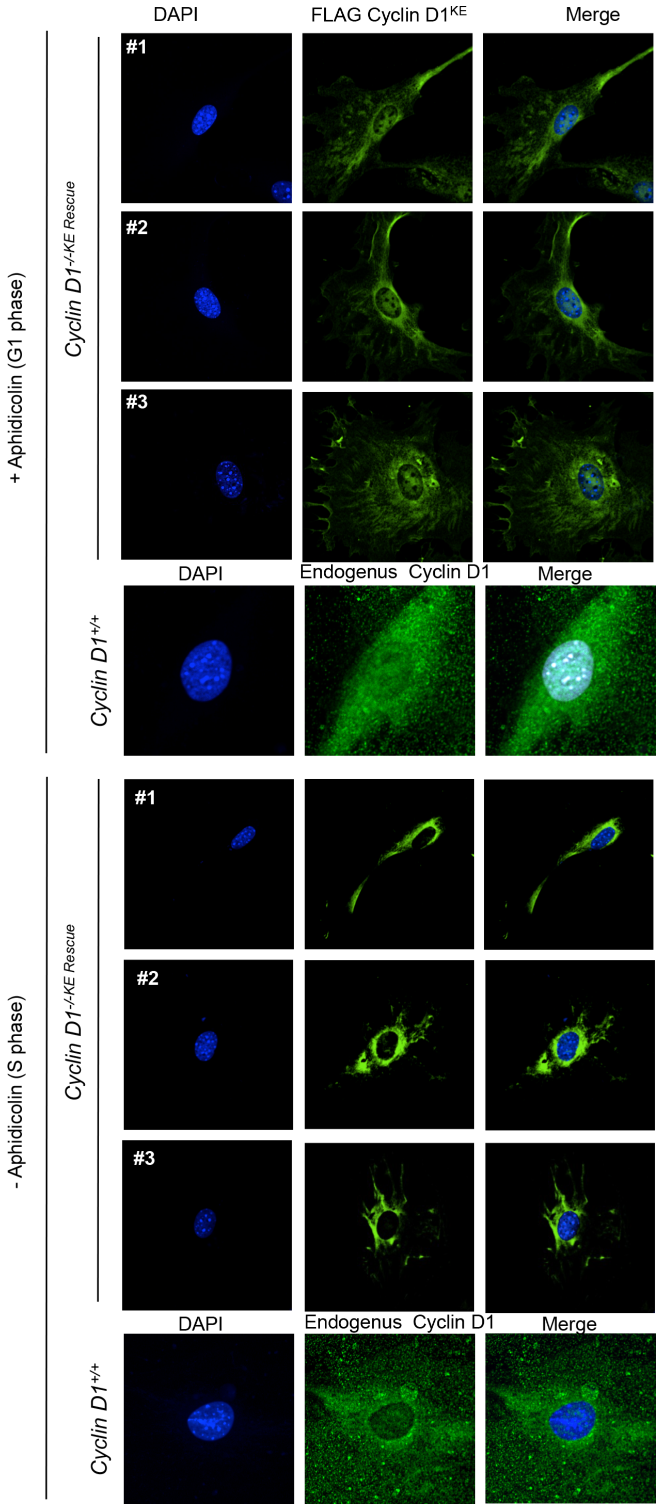

Supplementary Figure 2: Compartmentalization of exogenous cyclin D1^{KE} mimics endogenous cyclin D1 during transition from G1 to S phase. Three representative images of *cyclin D1$^{-/-KE Rescue}$* 3T3 fibroblast cells and one wild type 3T3 fibroblast cell arrested in G1 phase by treating with aphidicolin (+ Aphidicolin (G1 phase)) for 16 hrs then released for 6 hours into S phase (-Aphidicolin (S phase)). Cells stained for DAPI (blue) and anti-FLAG (green) for exogenous cyclin D1^{KE} and anti-cyclin D1 for endogenous cyclin D1.

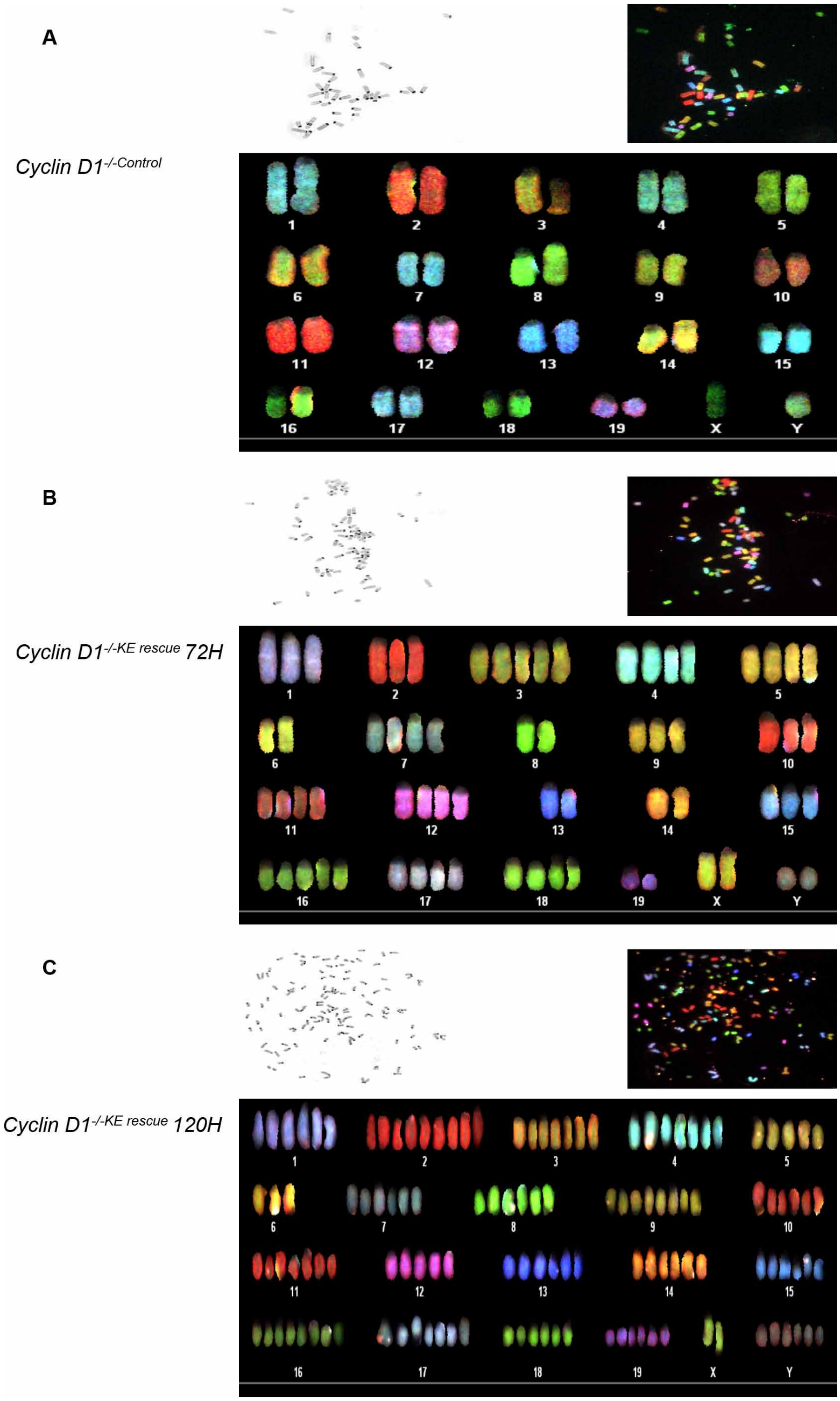

Supplementary Figure 3: Enlarged representative metaphases from Spectral karyotyping (SKY). Representative metaphases from Spectral karyotyping (SKY) on MEFs of *cyclin D1*$^{-/-Control}$ at 72 hours (P6) **(A)**, *cyclin D1*$^{-/-KE\ Rescue}$ at 72 hours (P6) **(B)** and *cyclin D1*$^{-/-KE\ Rescue}$ at 120 hours **(C)**. Each panel contains the following images: inverted 4′,6-diamidino-2-phenylindole (DAPI) image of the metaphase (top left corner), raw spectral image of the metaphase (top right) and classified metaphase of the same metaphase (lower panel).

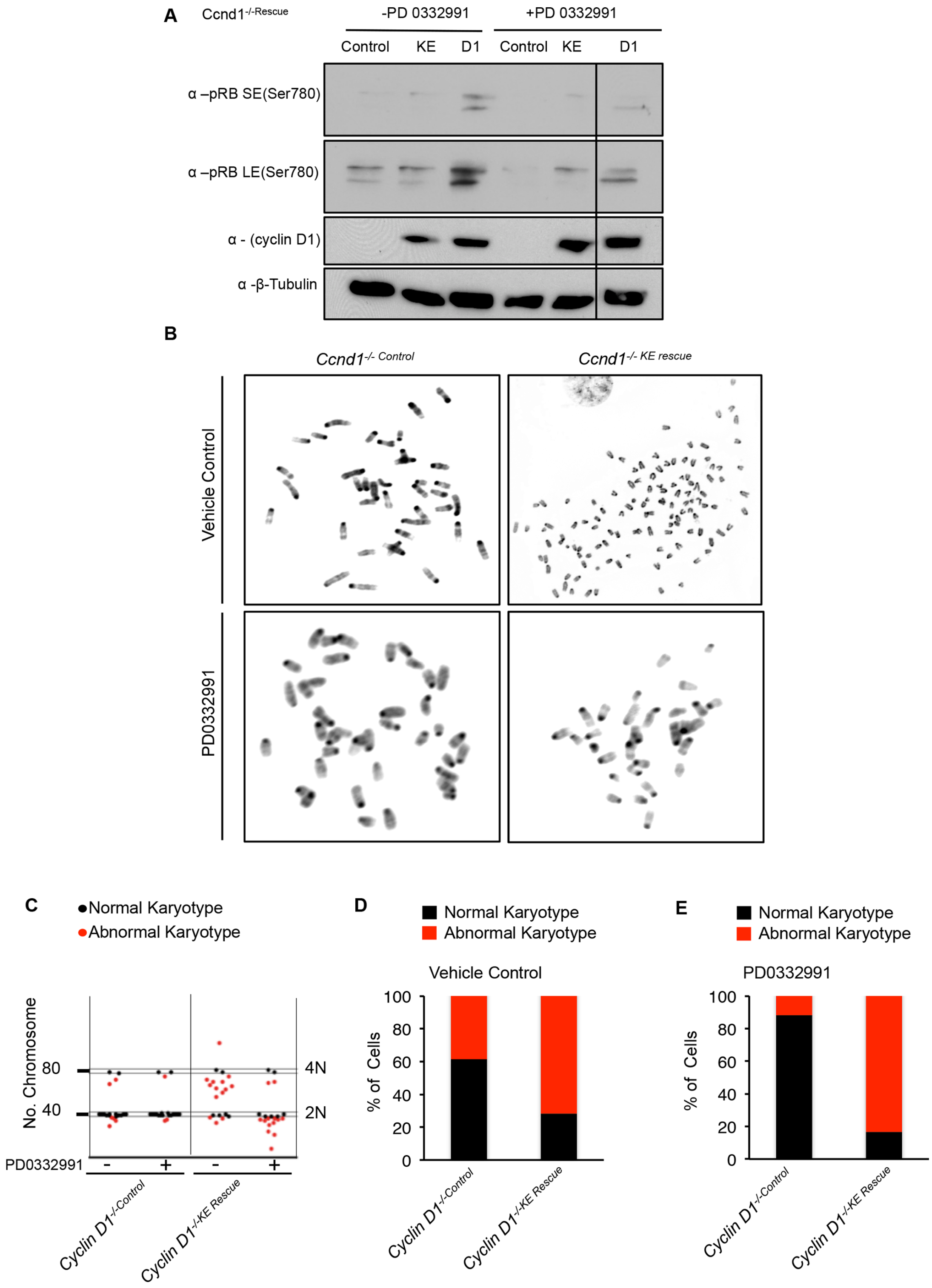

Supplementary Figure 4: Antagonist treatment of *cyclin D1$^{-/-D1\ Rescue}$* and *cyclin D1$^{-/-KE\ Rescue}$* MEF cells does not abrogate induction of aneuploidy. **(A)** Western blot demonstrating phosphorylation of RB (S780) and exogenous cyclin D1 abundance in *cyclin D1$^{-/-Control}$*, *cyclin D1$^{-/-KE\ Rescue}$*, MEF cells and *cyclin D1$^{-/-D1\ Rescue}$* 3T3 fibroblast cells treated with vehicle control or 1 µM CDK4/6 antagonist-PD0332991 for 72 hrs **(B)** Representative metaphases from *cyclin D1$^{-/-Control}$* and *cyclin D1$^{-/-KE\ Rescue}$* MEFcellstreated with control vehicle or CDK4/6 antagonist-PD0332991 for 72 hours. Metaphases were stained with DAPI and images converted to gray scale. **(C)** Scatter plots of chromosome numbers for *cyclin D1$^{-/-Control}$* and *cyclin D1$^{-/-KE\ Rescue}$* MEF cells treated with vehicle control or PD0332991. The parallel lines represent expected deviation from normal at 2N and 4N (+/- 2 chromosome) (n = 18 metaphases per group). **(D)** Bar graphs showing the number of normal and abnormal karyotypes comparing *cyclin D1$^{-/-Control}$* and *cyclin D1$^{-/-KE\ Rescue}$* MEF cells at 72 hours from (D) vehicle treated and **(E)** PD0332991 treated MEF cells.

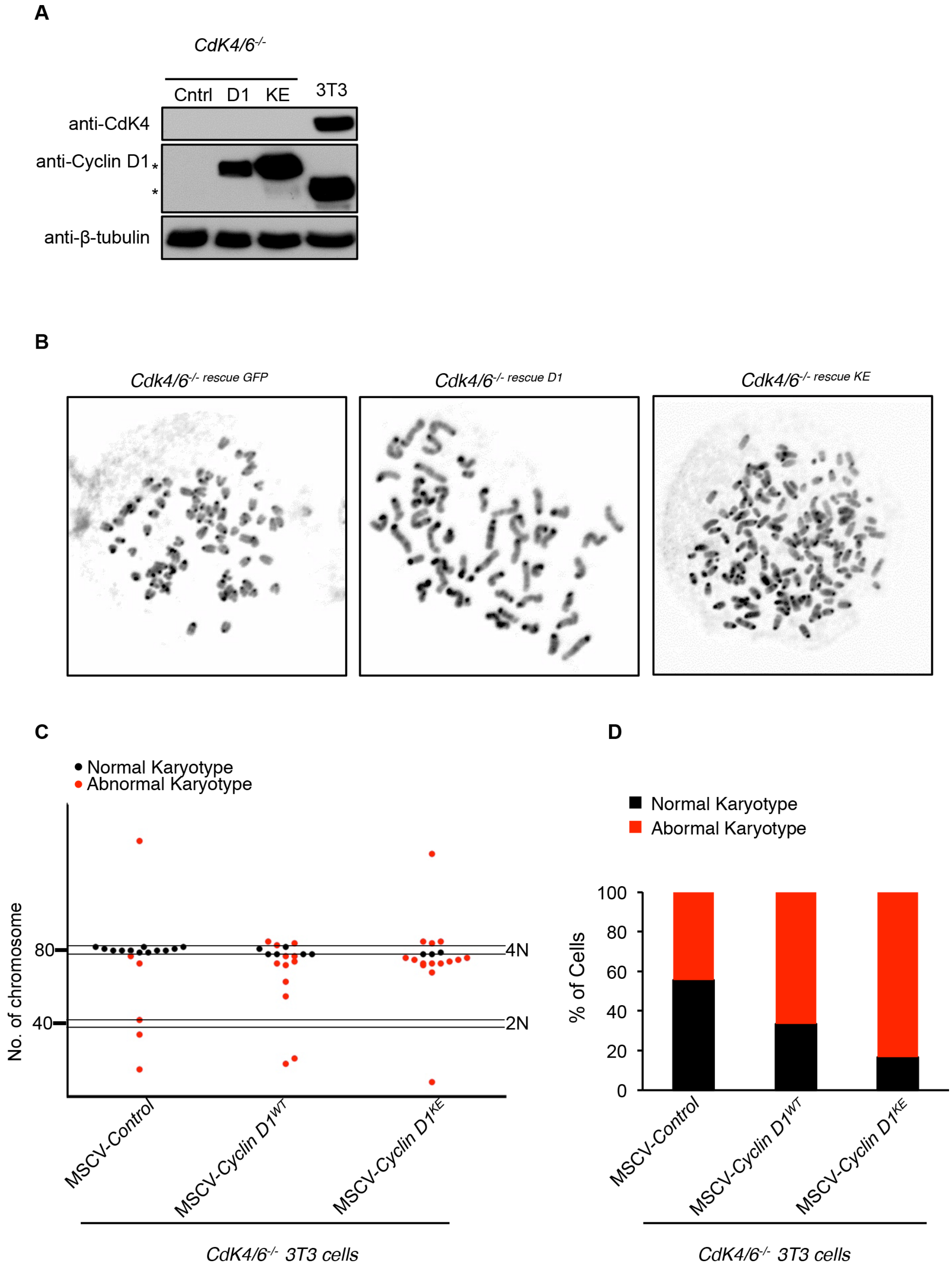

Supplementary Figure 5: Cyclin D1 induces aneuploidy in *cdk4/6*$^{-/-}$ 3T3 cells. (A)Western blot demonstrating cdk4 and exogenous cyclin D1 abundance in *cdk4/6*$^{-/-}$ 3T3 fibroblast cells transduced with MSCV-Control, MSCV-*Cyclin D1*^{WT} and MSCV-*Cyclin D1*^{KE}. 3T3 wild type fibroblast cells represent positive control for cdk4 abundance. **(B)** Representative metaphases from *cdk4/6*$^{-/-}$*/control, cdk4/6*$^{-/-}$*/cyclin D1*^{WT} and *cdk4/6*$^{-/-}$*/cyclin D1*^{KE} 3T3 fibroblast cells. Metaphases were stained with DAPI and images converted to gray scale. **(C)** Scatter plots of chromosome numbers for *cdk4/6*$^{-/-}$*/control, cdk4/6*$^{-/-}$*/cyclin D1*^{WT} and *cdk4/6*$^{-/-}$*/cyclin D1*^{KE} 3T3 fibroblast cells. The parallel lines represent expected deviation from normal at 2N and 4N (+/- 2 chromosome) (n = 15 metaphases per group). **(D)** Bar graph showing the number of normal and abnormal karyotypes comparing *cdk4/6*$^{-/-}$*/control, cdk4/6*$^{-/-}$*/cyclin D1*^{WT} and *cdk4/6*$^{-/-}$*/cyclin D1*^{KE} 3T3 fibroblast cells at 72 hours.

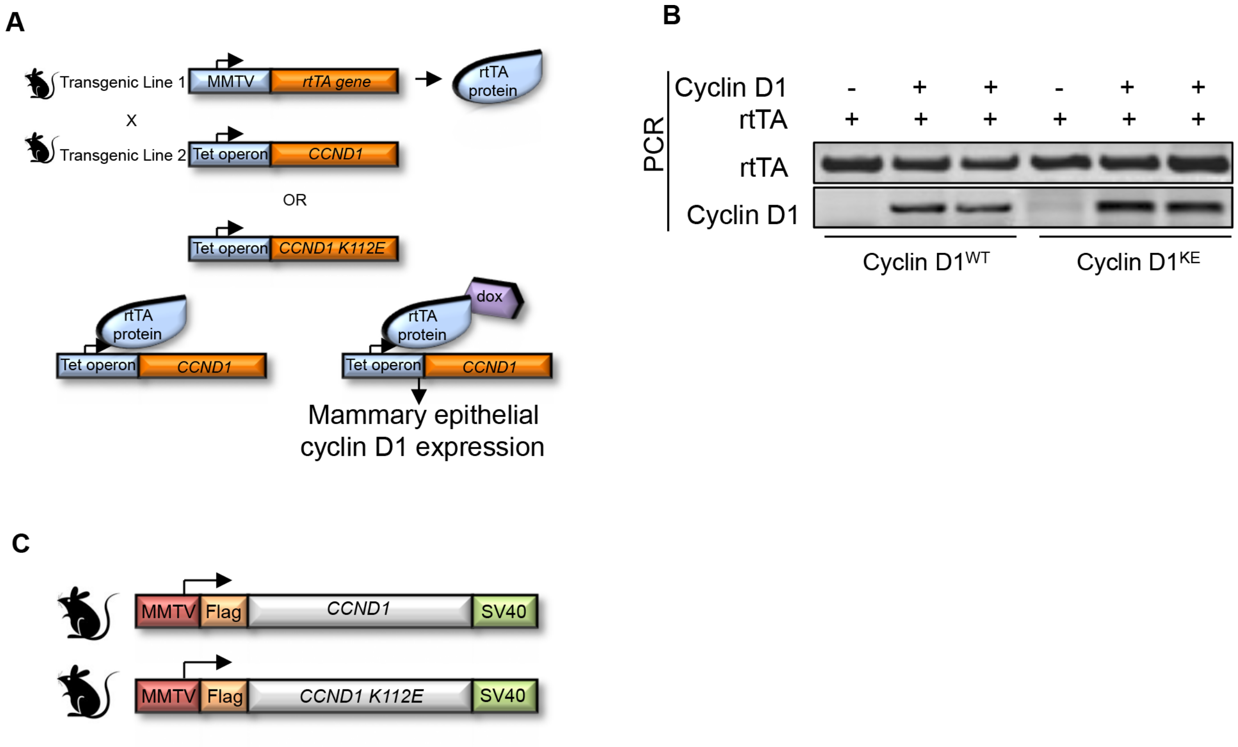

Supplementary Figure 6: Overview of Tet-*CCND1*^{WT} and Tet-*CCND1*^{KE} mice. (A)Schematic depiction of the tet-inducible mammary gland specific cyclin D1^{WT} and cyclin D1^{KE} transgenic lines. **(B)** Genotyping of Tet-*CCND1*^{WT}, Tet-*CCND1*^{KE}, and MMTV-rtTA transgenic mice. The cyclin D1^{WT} portion of figure has been published previously[20]. **(C)** Schematic depiction of the mammary gland specific MMTV-*CCND1*^{WT} and MMTV-*CCND1*^{KE} transgenic lines.

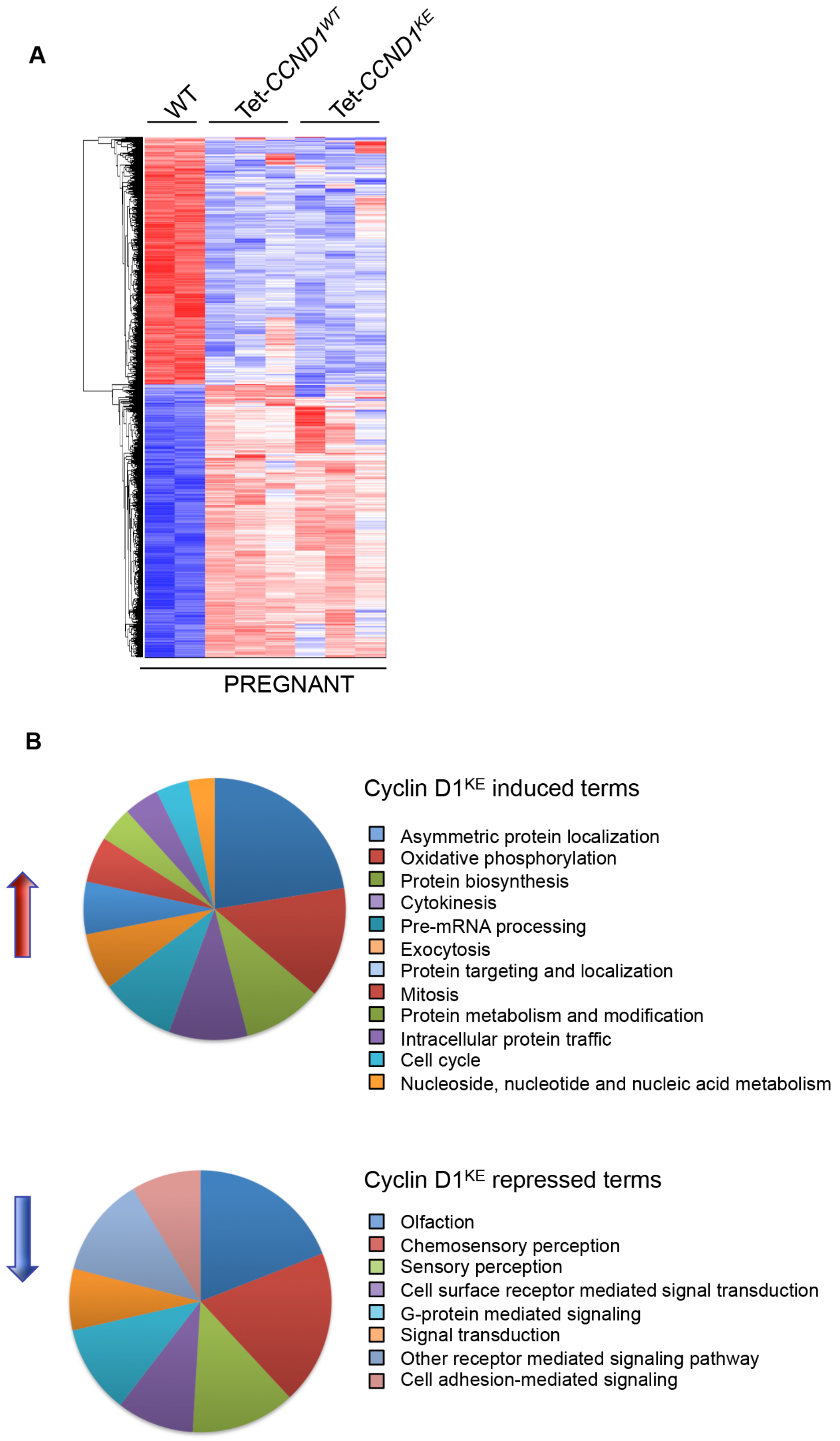

Supplementary Figure 7: Microarray analysis of Tet-*CCND1*^{WT} and Tet-*CCND1*^{KE} mouse mammary glands. **(A)** Hierarchical clustering of a superset of genes significantly differentially regulated between Tet-*CCND1*^{WT} vs control and Tet-*CCND1*^{KE} vs control mammary gland. **(B)** Pie chart representation of pathway analysis based on gene ontology (PANTHER-DAVID) of cyclin D1^{KE} induced and repressed genes charted using an enrichment score.

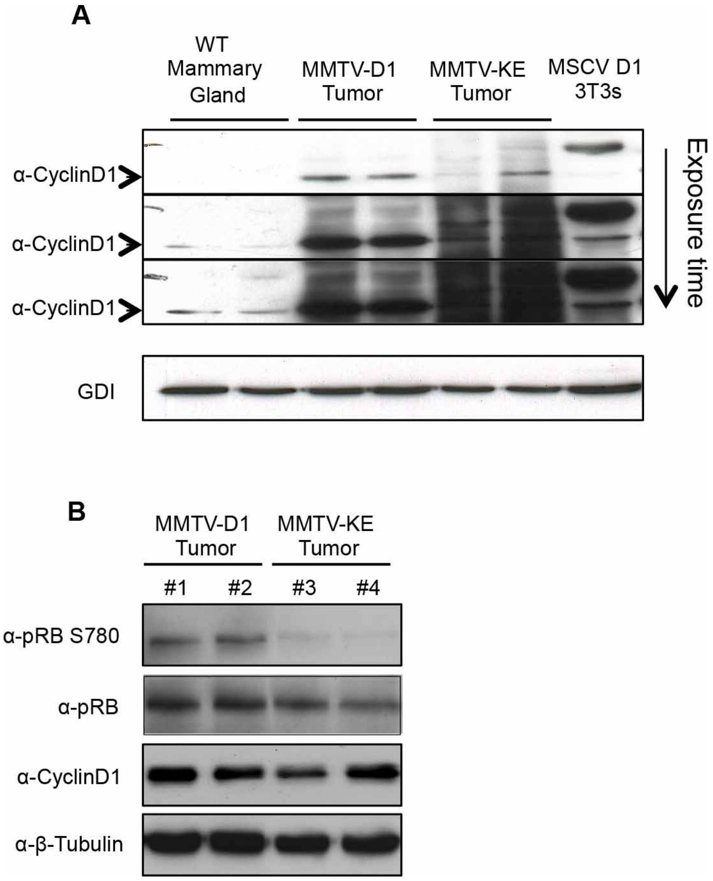

Supplementary Figure 8: Relative abundance of cyclin D1 and pRB phosphorylation status in mammary gland tumors derived from MMTV-*CCND1*^{WT} and MMTV-*CCND1*^{KE} mice. Western blot demonstrating **(A)** cyclin D1 abundance WT mammary gland compared to tumors from MMTV-*CCND1*^{WT} and MMTV-*CCND1*^{KE} mice. MSCV-cyclin D1^{WT} from 3T3 fibroblast cells used as a control; migration slightly retarded due to 3xFLAG. The WT mammary gland and MMTV-D1 tumor lanes have been published previously. **(B)** Western blot analysis of pRB S780 phosphorylation status in tumors from MMTV-*CCND1*^{WT} and MMTV-*CCND1*^{KE} mice.

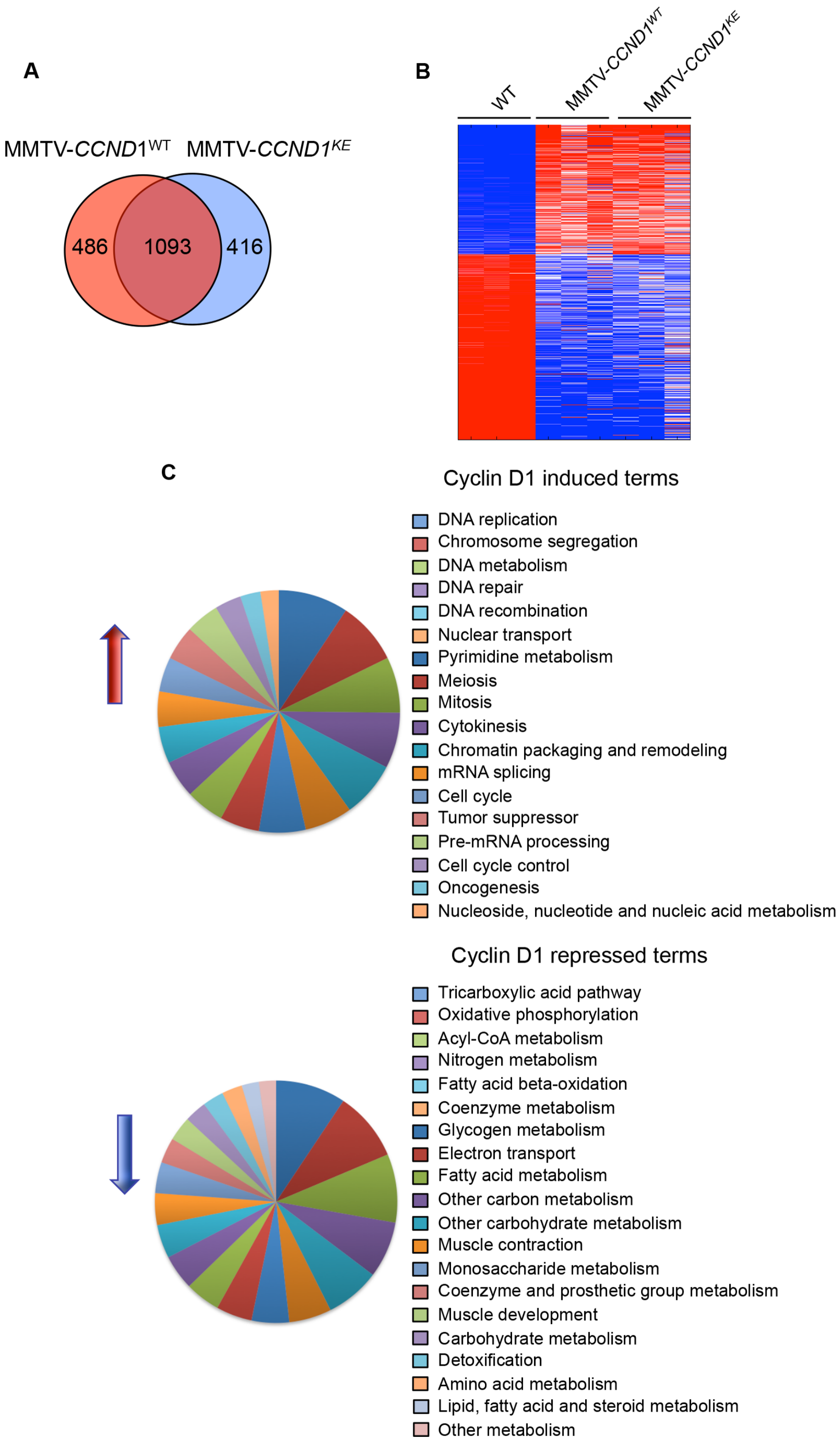

Supplementary Figure 9: Microarray analysis of MMTV-*CCND1^{WT}* and MMTV-*CCND1^{KE}* mouse mammary tumors. (A) Venn diagram representing genes differentially regulated by MMTV-*CCND1^{WT}* ($n = 3$) and MMTV-*CCND1^{KE}* ($n = 3$) and visualized by **(B)** Hierarchical clustering of 1995 significantly regulated genes ($P \leq 0.01$ and fold change > 2). **(C)** Pie chart representation of functional annotation clustering (DAVID) of cyclin D1^{KE} induced and repressed terms based on percentage enrichment score of the top hits.

Supplementary Table 1: Histological analysis of tumors from wild type FVB, MMTV-Cyclin D1WT and MMTV-Cyclin D1KE Mice

**WT FVB Mice (6 tumors)**
Adenocarcinoma with squamous differentiation
Papillary adenocarcinoma
Partially necrotic secretory adenocarcinoma
EMT-phenotype
Complex adenoacanthoma
EMT-phenotype
**MMTV-Cyclin D1^{WT} Mice (10 tumors)**
Keratoacanthoma
EMT-phenotype
Keratoacanthoma
Adenosquamous AC
Adenosquamous AC and Spindle cell tumor
Adenocarcinoma
Papillary Adenocarcinoma
Adenosquamous AC (Keratoacanthoma)
Lobular hyperplasia and secretory adenocarcinoma
EMT-phenotype
**MMTV-Cyclin D1^{KE} Mice (7 tumors)**
Partially necrotic adenocarcinoma
Adenocarcinoma
Papillary adenocarcinoma
EMT-phenotype
Complex adenoacanthoma
Papillary adenocarcinoma
EMT-phenotype

Supplementary Table 2: Interval data from cyclin D1^{KE} associated genomic regions generated by ChIP-Seq

**Description**	**Act Regions**
Number of Intervals Converted	4296
Number of Intervals within –10000/+10000 bp of NCBI Genes	3494
Number of Intervals NOT within –10000/+10000 bp of NCBI Genes	802
Number of NCBI Genes with Intervals within −10000/+10000 bp	4446
Number of Intervals within Promoter Region (−7500/+2500 bp of NCBI Gene Start)	3305
Number of NCBI Genes with intervals in Promoters (−7500/2500 bp of start)	3780

Supplementary Table 3: Full list of transcription factor binding sites identified in cyclin D1KE peak interval sequences

Transcription Factor Name	*P*-value	Experimental Hits
SP1	4.70E–07	1717
RREB1	2.64E–06	1233
GABPA	2.20E–08	810
EWSR1–FLI1	9.78E–07	805
ETS1	3.82E–07	729
ELK4	7.83E–08	551
FEV	8.19E–08	521
NR2F1	2.41E–06	512
MZF1_1–4	3.06E–07	489
CTCF	1.29E–07	483
ZFX	1.90E–07	477
ELK1	8.06E–08	471
GATA2	7.40E–07	471
NFYA	6.12E–08	422
MYF	6.45E–08	418
PLAG1	5.06E–07	384
ZNF143	4.06E–07	382
EGR1	2.25E–08	378
ZEB1	1.29E–07	351
IRF1	1.85E–06	343
ARNT	6.21E–07	337
USF1	6.21E–07	337
NR4A1	1.03E–06	316
PAX5	1.50E–07	304
CREB1	9.10E–08	299
STAT3	3.15E–07	294
SPIB	3.76E–08	290
NHLH1	4.61E–07	289
ESR1	1.65E–07	274
PPARG::RXRA	6.42E–08	274
SPI1	5.73E–07	267
STAT1	1.51E–07	261
REST	5.00E–08	255
FOXD3	1.09E–07	244
MZF1_5–13	1.51E–07	233
NFIC	7.09E–08	219

(*Continued*)

Transcription Factor Name	*P*-value	Experimental Hits
MAX	1.02E–07	217
YY1	1.41E–07	204
ESR2	2.57E–07	186
MYCN	7.59E–08	176
REL	7.61E–08	174
ESRRB	7.10E–07	168
HNF4A	2.37E–07	165
RUNX1	1.80E–08	161
NFE2L1::MAFG	6.41E–07	157
FOXA1	2.92E–07	144
RXR::RAR_DR5	4.72E–08	141
HAND1::TCFE2A	1.00E–08	140
NFKB1	6.46E–08	137
ARID3A	1.07E–06	136
FOXO3	1.37E–07	136
MYC::MAX	2.33E–07	132
PAX2	1.95E–08	130
TP53	3.05E–07	121
NF–KAPPAB	2.05E–08	120
NR2E3	2.99E–08	120
IRF2	1.00E–08	118
GFI	2.75E–07	117
KLF4	4.63E–07	116
SPZ1	9.76E–08	115
RELA	7.28E–08	112
MAFB	3.95E–08	111
PPARG	1.00E–08	111
TAL1::TCF3	4.13E–02	108
MYC	6.75E–08	107
AP1	8.31E–08	106
NFE2L2	1.25E–05	103
AR	1.00E–08	101
FOXA2	1.12E–05	101
MEF2A	2.24E–07	101
E2F1	2.36E–06	99
NFATC2	1.59E–05	90
INSM1	2.47E–03	89

(*Continued*)

Transcription Factor Name	*P*-value	Experimental Hits
NR1H2::RXRA	4.96E–04	88
RORA_1	1.41E–07	88
EVI1	1.29E–02	87
SOX2	1.19E–04	87
SRY	2.12E–07	87
RXRA::VDR	7.53E–07	86
GATA3_1	1.00E–08	81
NR3C1	1.27E–07	80
GATA1	2.60E–07	78
SOX5	2.23E–06	78
NKX2–5	2.00E–06	76
FOXF2	7.68E–03	75
DDIT3::CEBPA	4.84E–04	73
MIZF	1.00E–08	72
FOXL1	1.17E–05	66
MYB	1.04E–07	66
SOX17	8.70E–03	65
HIF1A::ARNT	4.34E–04	64
HNF1A	2.39E–03	63
LHX3	1.90E–02	62
NKX3–2	8.93E–04	62
CEBPA	2.73E–08	61
PAX6	3.81E–03	59
TAL1::GATA1	4.12E–04	59
SOX9	5.15E–06	58
FOXC1	3.81E–05	55
HLF	6.66E–08	54
NOBOX	1.07E–06	54
FOXD1	2.14E–05	53
HNF1B	2.61E–06	53
RORA_2	3.55E–04	52
SRF	1.35E–04	51
T	2.91E–02	51
BRCA1	8.58E–05	48
PAX4	1.34E–04	48
PRRX2	2.62E–03	45
HOXA5	1.90E–02	43
PDX1	7.33E–03	39
HLTF	3.41E–02	35

Supplementary Dataset 1: Full list of genes regulated by cyclin D1^{WT} and cyclin D1^{KE} in mammary gland of transgenic mice

Supplementary Dataset 2: Full gene list of the mammary tumors for MMTV-cyclin D1^{KE} and MMTV-cyclin D1^{WT} mice, includes list of genes common to the two sets

Supplementary Dataset 3: Complete list of cyclin D1^{KE} bound genes by ChIP-Seq analysis
